# HLA Class I and II Alleles in Anti-Acetylcholine Receptor Antibodies Positive and Double-Seronegative Myasthenia Gravis Patients of Romanian Descent

**DOI:** 10.3390/neurolint16060130

**Published:** 2024-12-10

**Authors:** Cristina Georgiana Croitoru, Daniela Constantinescu, Mariana Pavel-Tanasa, Dan Iulian Cuciureanu, Corina Maria Cianga, Diana Nicoleta Hodorog, Petru Cianga

**Affiliations:** 1 Neurology Clinic I, “Prof. Dr. Nicolae Oblu” Emergency Clinical Hospital, 700309 Iași, Romania; 2 Department of Immunology, “Grigore T. Popa” University of Medicine and Pharmacy, 700115 Iași, Romania; 3 Immunology Laboratory, “St. Spiridon” Hospital, 700111 Iasi, Romania; 4 Medical Department III-Neurology, “Grigore T. Popa” University of Medicine and Pharmacy, 700115 Iași, Romania

**Keywords:** Human Leukocyte Antigen (HLA), myasthenia gravis, etiopathogenic mechanisms, anti-acetylcholine receptor, double-seronegative

## Abstract

**Background**: Several significant associations between certain Human Leukocyte Antigen (HLA) alleles and myasthenia gravis (MG) subtypes were established in populations from Western Europe and North America and, to a lesser extent, from China and Japan. However, such data are scarcely available for Eastern Europe. This study aimed to analyze the associations of HLA Class I and II alleles with MG and its serological subtypes (with anti-acetylcholine receptor autoantibodies, RAch+MG, and double-seronegative, dSNMG) in myasthenic patients of Romanian descent. **Methods:** We consecutively enrolled adult Romanian unrelated myasthenic patients, which were genotyped by next-generation sequencing for HLA-A, -B, -C, -DRB1 and -DQB1. The descent-matched controls were represented by two separate groups of random normal subjects genotyped for the main five HLA loci at the two-digit and four-digit levels, respectively, collected from the Allele Frequency Net Database. **Results:** A total of 40 patients (females: 80.00%; median age at onset: 42.5 years, range: 1–78; RAch+MG: 75.00%; dSNMG: 22.50%) were included. We were able to confirm previously acknowledged allelic associations: positive for HLA-B*08, DRB1*14:54 and DRB1*16:01 and negative for DRB1*13. However, we found some potential novel significant positive associations between MG and the HLA-A*02:36, B*47, B*73, B*44:27 and B*57:02 alleles. All alleles positively associated with MG remained significantly associated with RAch+MG, regardless of the patients’ clinical and thymic heterogeneity. We found significant positive associations between dSNMG and the HLA-B*47, B*44:27 and DRB1*14:54 alleles that are shared with RAch+MG. **Conclusions:** These results suggest both distinct and common etiopathogenic mechanisms between dSNMG and RAch+MG. Our study pioneers allele associations in Romanian MG patients.

## 1. Introduction

Myasthenia gravis (MG), the most frequent autoimmunity of the neuromuscular junction, with a prevalence of 150–200 cases per million [1], is a postsynaptic disease caused by autoantibodies directed most frequently against the acetylcholine receptor (RAch) in up to 90% of the generalized cases and against the muscle-specific receptor tyrosine kinase (MuSK) in 1–10% of cases [1,2,3]. Double-seronegative MG (dSNMG), defined by the absence or low-affinity of these two autoantibodies, is a rarer subtype, most likely caused by antibodies against still unidentified targets [1]. Hence, the functional disruption at the nerve–muscle interface causes effort-dependent fatigability in various unsystematized skeletal muscle groups [4]. The main generally acknowledged clinical subtypes are ocular (OMG) vs. generalized (GMG) and early-onset (EOMG) before 50 years of age vs. late-onset (LOMG) [1].

Unlike the well-understood immunopathogeny of this T cell-dependent B cell-mediated disease, the etiology of MG remains an open canvas, as in many other multifactorial autoimmune conditions [5]. The concordance rate among monozygotic twins is much less than 100%. It is however higher than the one among dizygotic twins, and this allows us to consider MG as a disease caused, to some extent, by genetic factors [6]. The HLA (Human Leukocyte Antigen) genes, and thus the proteins of the human Major Histocompatibility Complex (MHC), seem to play a major etiopathogenic role. This theory is supported by many studies performed within the last decades, including four genome-wide association studies (GWAS) that established several significant associations between certain HLA alleles and MG subtypes [7,8,9,10]. These associations may play an important role in the diagnosis and treatment of MG, as they also provide insights regarding the immune-mediated mechanisms of the disease. For instance, certain individuals at risk for developing MG or with an unconfirmed diagnosis may benefit from HLA genotyping in order to test the presence of certain alleles known as risk factors. Taking into consideration the well-established role of MHC class II molecules in autoreactive T cells recognition of the autoantigen and the multiple associations between MG and HLA class II alleles, there are currently studied therapeutical molecules that specifically block the MHC class II molecules’ antigen binding site [11]. Among the most notable advantages of this therapeutical approach is the targeted immunosuppression, which avoids the generalized one produced by current anti-myasthenic treatments. Furthermore, the targeted immunosuppression offers, at least theoretically, the possibility of reducing in time the number of autoreactive T cells [11]. In other words, although HLA genotyping is not a routine test for myasthenic patients according to worldwide accepted protocols; in the future, it may become a screening method and a triage tool for administering potentially curative anti-myasthenic treatments.

In Caucasian populations, EOMG is strongly linked with the ancestral haplotype A*01-B*08-C*07-DRB1*03:01-DQB1*02:01 [10,12,13], while LOMG is associated with HLA-DRB1*01 [14], HLA-DRB1*14 [15], HLA-DRB1*16 [16] and HLA-DRB1*15:01 alleles [17]. HLA-DRB1*14 [18] and HLA-DRB1*16 [19] alleles are also associated with MuSK-positive MG. Few studies report associations between MG with thymoma and HLA-A*03, HLA-A*24 [20] and HLA-A*25 [21], but these associations were not confirmed by other studies. Overall, no allelic associations were reported for dSNMG [1]. In spite of all these allelic associations, the exact extent of the cause–effect relationship is yet to be discovered as we are facing two major impediments: the complexity of the HLA super-locus, characterized by a tremendous polymorphism, but by strong linkage disequilibrium as well on one side [22], and the heterogeneous nature of MG, on the other side.

However, the vast majority of the associations between various HLA alleles and MG were reported in patients predominantly from Western Europe, Northern Europe and North America, while few studies were performed on populations of Eastern European descent. Regarding the latter origin, after typing the DRB1* and DQB1* loci by polymerase chain reaction reverse sequence-specific oligonucleotide probe in 31 MuSK-positive myasthenic Serbian patients, Nikolic et al. discovered significant associations with the DQB1*05, DRB1*14 and DRB1*16 alleles [23]. According to a study performed on 217 Ukrainian myasthenic patients, the DR5 phenotype has a higher incidence among MG cases with both normal thymus and thymic hyperplasia, while the DR1 phenotype has a higher frequency among the latter, and 89% of HLA-DR7 carriers have thymomatous MG [24].

Given the ethnical heterogeneity of the Romanian population as a result of its particular origin, modeled by numerous geopolitical influences and migrations, one can deduct the complexity of the Romanians’ genetical changes, including the HLA genomic region. Since no allelic associations were reported so far in myasthenic patients of Romanian descent, a genetic study dedicated to this particular subject may have the potential not only to provide a different perspective of the pathogeny of this disease, but also to offer a scientific basis in implementing novel specific anti myasthenic treatments in these patients.

The present case–control study aimed at analyzing the associations of HLA Class I (A, B, C) and II (DRB1, DQB1) alleles with MG and its serological subtypes (RAch+MG, dSNMG) in 40 unrelated myasthenic patients of Eastern European Romanian descent.

## 2. Materials and Methods

### 2.1. Patient Population and Data Collection

To study a rare disease, we included all patients diagnosed with MG who were registered at our regional neurology hospital during the three-year study period. This approach was taken to ensure sufficient power for identifying relevant associations. Therefore, from 112 confirmed cases of MG admitted in the First Neurology Clinic of the “Prof. Dr. N. Oblu” Emergency Clinical Hospital, Iași, Romania, between 01.01.2020 and 31.12.2022, we enrolled in our study a total of 40 consecutive patients who met all the inclusion criteria and none of the exclusion ones. The inclusion criteria were as follows: (1) positive MG diagnosis; (2) age at admission ≥ 18 years; (3) Romanian ancestry; (4) lack of kinship; (5) written consent to participation. In accordance to international guidelines, a positive MG diagnosis was assigned only when the clinical criteria (clinically objectified effort-dependent fatigability in any of the cephalic extremity, limb, axial, respiratory skeletal muscles and a positive neostigmine test) associated serological criteria (high serum levels of either AchR or MuSK antibodies) and/or electrophysiological criteria (over 10% decrement of the compound muscle action potential between the first and the fifth stimulus at slow rate electrical stimulation of the nerve and/or prolonged jitter at single-fiber electromyography) [1]. The major cause of exclusion was refusal of participation, as 71 patients would not sign the written consent. In only one case, we encountered a technical error during HLA typing that required obtaining a new blood sample. Since the patient refused the procedure, we cataloged the case as withdrawn from the study.

The main clinical and paraclinical variables of interest collected both retrospectively from the patients’ electronic charts and prospectively by anamnesis, clinical consult and laboratory tests were gender, age at onset, MG Foundation of America (MGFA) class, autoimmune comorbidities, thymic pathology, main pathogenic antibodies status (RAch, MuSK) and anti-titin and anti-ryanodine receptor calcium release channel (RRCRC) antibodies status.

We used MGFA classes to segregate the patients into OMG (class I) and GMG (classes II–V) [25]. In all cases, thymic pathology was assessed initially by chest computer tomography (CT) and by thymus histology whenever thymectomy for thymoma or thymic hyperplasia was performed (15 out of 40).

### 2.2. Controls

The source for the allelic frequencies of the descent-matched controls was the Allele Frequency Net Database (www.allelefrequencies.net, accessed on 20 October 2023). For analyzing allelic associations at low-resolution level (two digits), we used a control population reported by Constantinescu et al., corresponding to Romaniapop2 in the database. This control group consists of random normal Romanian subjects genotyped for the HLA-A (n = 5758), B (n = 6010), C (n = 1612), DRB1 (n = 6936) and DQB1 (n = 3328) loci. For analyzing allelic associations at high-resolution level (four digits), we used a second control population of 1234 random normal subjects of Romanian descent genotyped for HLA-A, B, C and DRB1 loci. This population was reported by Pingel J. et al. [26] and corresponds to Germany DKMS Romanian minority in the Allele Frequency Net Database.

In addition, we compared the frequencies of the alleles identified in our myasthenic patients with their estimated frequencies in Romania [27]. The data provided for Romania in the allele frequency database are relatively recent, covering the past 10 years for HLA genomic analysis. Thus, the bias associated with differing time points of investigation between the two groups, MG and controls, was minimized.

### 2.3. Detection of Anti-Titin, Anti-Ryanodine Receptor Calcium Release Channel, Anti-Acethylcholine Receptor and Anti-Muscle-Specific Receptor Tyrosine Kinase Antibodies

Serum anti-titin and anti-RRCRC antibodies were detected by indirect enzyme-linked immunosorbent assays, using two distinct qualitative commercial ELISA kits (AFG Bioscience, Northbrook, IL, USA). Frozen aliquots of the patients’ sera were stored at −20 °C until tested, according to the manufacturer’s instructions. The ELISA plates were incubated at 37 °C, in a Sanofi Diagnostic Pasteur (France) plate incubator. The 450 nm absorbance was measured in each well with a Tecan Infinite M Plex (Austria) spectrophotometer, and the results were generated using Magellan v7.4 (Austria) software.

The anti-MuSK autoantibodies were detected by ELISA with the TECAN RE51021 MuSKAb quantitative kit (IBL International GmbH, Hamburg, Germany). According to the manufacturer’s instructions, the plates were incubated at 18–25 °C, and the optical density was measured at 405 nm. The results were assessed by reporting to the cut-off control index.

The detection of the anti-RAch autoantibodies was performed by radio immune assay (RIA) using the RiaRSRTM AchRAb commercial kit (RSR Limited, Cardiff, UK). The frozen sera aliquots were brought to room temperature and incubated with a mixture of adult and fetal forms of the receptor and labeled with 125I-labeled alpha bungarotoxin. The autoantibodies binding to RAch generated immune complexes that were further precipitated with anti-human IgG. The precipitate was isolated by centrifugation, and its radioactivity was counted with a gamma counter.

### 2.4. HLA Genotyping

Genomic DNA was extracted from peripheral blood, harvested in EDTA vacutainers using the BioMagPure Blood DNA Extraction Kit 200 (Zinexts Life Science Corporation, New Taipei City, Taiwan) on a BioMagPurix 12 Plus system (Zinexts Life Science Corporation, New Taipei City, Taiwan). Amplification and library preparation were performed with the NGSgo℗ MC6-1 and NGSgo℗ Library Full kit GenDx reagents (GENDX, Utrecht, The Netherlands). The NGSgo library was further sequenced on an iSeq Illumina genomic sequencer (Illumina, San Diego, CA, USA), and the sequences were analyzed using GENDX NGSengine software (GENDX, Utrecht, The Netherlands) version 2.21.0.20156.

### 2.5. Statistical Analysis

The statistical analysis was performed using Statistical Program for Social Sciences software (SPSS) (version 18, IBM Corporation, New York, NY, USA) and EXCEL (version 1811, Microsoft Office LTSC Professional Plus 2021).

Continuous variables were reported as the mean (±standard deviation, SD) or median, while categorical variables were as numbers and percentages. The chi-square test was used for comparing categorical variables.

Within the patients’ group, we calculated the HLA allele frequencies by counting each type, twice for the homozygous cases. We used the proportion Z test for initially comparing the allelic frequencies of the myasthenic patients with their corresponding counterparts in the entire Romanian population. When comparing the allelic frequencies between the MG group and the two control groups, we performed a 2 × 2 contingency table analysis using the chi-square test, with Yates correction in the case of rare alleles (n < 5). We assessed the strength of the significant associations by odds ratio (OR) and 95% confidence intervals (CIs). For allelic associations, we corrected the *p*-values for multiple comparisons using the Benjamini–Hochberg method [28]. The Hardy–Weinberg equilibrium was applied, using the chi-square test, as explained by Chen et al. [29]. The linkage disequilibrium (LD) was computed as the difference between the observed frequency of a given haplotypes (PXY) and the theoretical expected frequency (PX × PY). *p*-values less than 0.05 were considered statistically significant.

## 3. Results

### 3.1. General Characteristics of Myasthenia Gravis Patients

Among the 40 patients studied, women represented the majority, with a female:male ratio of 4:1. With an age at onset ranging from 1 to 78 years, the study group included 23 EOMG cases, 14 LOMG cases and 3 juvenile MG (MGj) cases. As the latter were represented only by females, we further stratified them by the presence of menarche in pre-pubertal (1) and post-pubertal (2) onset. Patients with GMG outnumbered the OMG patients by seven. The main clinical and serological features are summarized in Table 1.

Autoimmune comorbidities were represented by isolated chronic autoimmune thyroiditis (7), isolated rheumatoid arthritis (1), association between the aforementioned (1) and autoimmune hepatitis (2). All co-occurring autoimmunities were encountered exclusively in women and in all MGj cases. All three patients with anti-titin antibodies, out of which two also tested positive for RyR antibodies, were females diagnosed with GMG (2 LOMG and 1 EOMG); they had no autoimmune comorbidities, and their thymus had a normal CT aspect. Both post-pubertal onsets MGj cases were RAch+, while the one with a prepubertal onset was dSNMG.

### 3.2. Allelic Frequencies in Myasthenia Gravis Cases vs. Controls

By HLA genotyping for the HLA-A, B, C and DRB1 loci of 40 patients and for the HLA-DQB1 loci of 39 patients, we thus recorded 95 alleles: 16 HLA-A, 30 HLA-B, 17 HLA-C, 19 HLA-DRB1 and 13 HLA-DQB1.

When comparing the frequencies of the alleles detected in our patients group with their corresponding counterparts in the entire Romanian population, we discovered that in the myasthenic group alleles B*08 (15% vs. 7.70%, *p* = 0.014), B*40 (10% vs. 5%, *p* = 0.040), B*47 (2.50% vs. 0.60%, *p* = 0.028), B*73 (1.25% vs. 0.10%, *p* = 0.001) and DRB1*16 (17.50% vs. 9.60%, *p* = 0.017) were significantly more frequent, while alleles A*11 (2.50% vs. 9.30%, *p* = 0.036) and DRB1*13 (1.25% vs. 10.50%, *p* = 0.007) were significantly less frequent.

When comparing allele frequencies between our myasthenic cases and the control group (Romaniapop2), at the two-digit level, we found positive associations at HLA-B, DRB1 and DQB1 loci between the following alleles and MG: the strongest were with the alleles B*47 and B*73, followed by B*08, DRB1*16 and DQB1*05, while the weakest was with B*40. A negative association emerged for DRB1*13. However, after correction, only associations with the B*47, B*73, B*08 and DRB1*13 alleles remained significant (Table 2).

Since HLA-B*40 presented a weak, however novel positive association with MG, with a significantly higher frequency in the myasthenic patients when compared to the general population of Romania, we next compared clinical and thymic features between carriers (n = 8) and non-carriers (n = 32) in order to investigate the possibility whether this allele could represent a disease biomarker (Table 3). The only significant difference that emerged was regarding autoimmune comorbidities, which were more frequent in the B*40 carriers.

When comparing allele frequencies between our myasthenic cases and the control group (Germany DKMS Romanian minority), at the four-digit level, after correction for multiple testing, we found a total of five positive associations at HLA-A*, B* and DRB1* loci which also maintained a statistically significant OR (Table S1). The strongest association were with DRB1*14:54 allele (6.25% vs. 0.00%, *p*c = 0.00048, OR (95% CI) = 359.6490 (19.7070–6563.5224)) and B*47:27 (2.50% vs. 0.00%, *p*c = 0.00064, OR (95% CI) = 157.2293 (7.4856–3302.4752)), followed by B*57:02 (2.50% vs. 0.08%, *p*c = 0.0032, OR (95% CI) = 31.6154 (4.3961–227.3664)), DRB1*16:01 (16.25% vs. 6.44%, *p*c = 0.0144, OR (95% CI) = 2.8177 (1.5228–5.2137)) and A*02:36 (1.25% vs. 0.00%, *p*c = 0.0312, OR (95% CI) = 93.1509 (3.7651–2304.6180)). We also discovered negative associations between MG and multiple alleles of the A, B and DRB1 loci; however, their respective OR was not significant (Table S1).

### 3.3. Allelic Frequencies in RAch+MG and dSNMG Cases vs. Controls

We next divided our 40 patients according to their serotype. Since our patients’ group included only one case of MuSK+MG, we analyzed the existence of possible associations between HLA alleles and, separately, RAch+MG (n = 30) and dSNMG (n = 9) by comparing the allelic frequencies (at the two-digit and four-digit levels) of each subgroup with the controls (Romaniapop2, respectively, Germany DKMS Romanian minority).

In the dSNMG subgroup, at the two-digit level genotyping we discovered only one significant positive association with B*47 allele and no negative ones (Table 2). At the four-digit level, we found two significant positive associations with B*44:27 (5.55% vs. 0.00%, *p*c = 0.000064, OR (95% CI) = 423.1714 (16.6558–10,751.4466), *p* = 0.0002) and DRB1*14:54 (11.11% vs. 0.00%, *p*c = 0.00006, OR (95% CI) = 748.0303 (34.5591–16,191.0695), *p* < 0.0001) (Table S2). The HLA-A, B, C and DRB1 alleles that were negatively associated with dSNMG and had a significant OR are detailed in Table S2. All these alleles were rare in the control group (frequencies between 0.04–0.16%) and absent in the dSNMG group.

RAch+MG, at the two-digit level and after correction, was positively associated with B*08, B*47 and B*73 and weakly negatively linked with DRB1*13 (Table 2). At the four-digit level, after correction, we found significant positive associations with the same alleles as for total MG, though with a stronger significance for A*02:36, B*44:27, B*57:02 and DRB1*16:01 and a similar significance with DRB1*14:54 (Table 4). We also discovered multiple negative associations between MG and alleles of the A, B and DRB1 loci; however, their respective OR was not significant (Table 4).

### 3.4. HLA-A, HLA-B, and HLA-DRB1 Haplotypes in MG Cases vs. Controls

Next, we aimed to examine the differences in HLA-A or HLA-B or HLA-DRB1 haplotypes’ distribution between MG and control cases from the initial Romanian population group. The top three most frequent HLA-A haplotypes observed for MG were A*02-A*24 (15%), A*02-A*02 (12.5%), and A*01-A*02 (10%), while, for the controls, were A*01-A*02 (10.46%), A*02-A*02 (6.97%) and A*02-A*03 (6.43%). Interestingly, the following HLA-B haplotypes were noticed only in the MG group: B*08-B*55, B*08-B*73 and B*13-B*27. For HLA-DRB1 haplotypes, the most frequent were DRB1*03-DRB1*16 (10%), DRB1*11-DRB1*16 (10%) and DRB1*04-DRB1*16 (7.5%) for MG and DRB1*07-DRB1*11 (5.22%), DRB1*11-DRB1*16 (5.22%) and DRB1*11-DRB1*15 (4.48%) for the controls. Applying the Hardy–Weinberg equilibrium test, HLA-A and HLA-B haplotypes were in disequilibrium for both controls and MG cases, while the HLA-DRB1 haplotypes only for the MG group (Table 5). We next conducted the linkage disequilibrium analysis and confirmed a disequilibrium related to HLA-A*02-A*24, A*01-A*03 and A*01-A*02 and for the previously identified HLA-DRB1 haplotypes; for the HLA-B haplotypes, a LD coefficient value above 4 was observed for B*40-B*47 and B*07-B*35 in the MG group and a value above 2 for the B*15-B*35 haplotype in the control group (Table S3).

**Figure 1:**
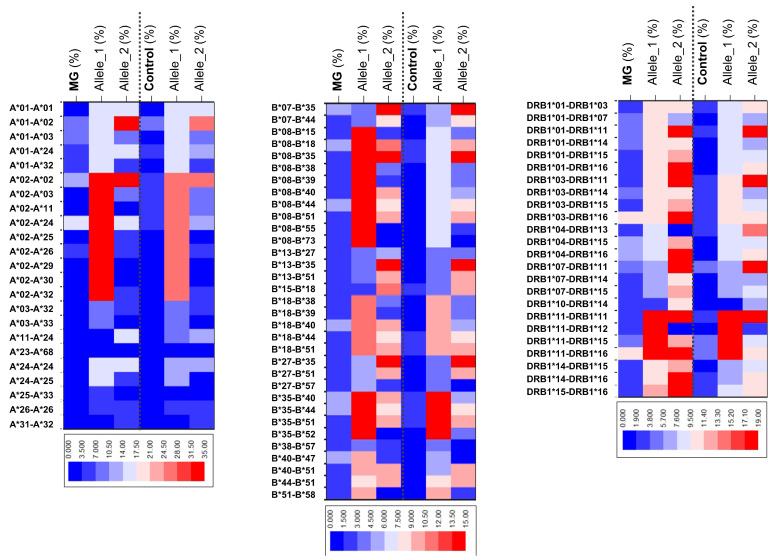
Heatmap of frequency distribution of HLA haplotypes and individual alleles among MG and control cases.

A heatmap displaying the frequency of haplotypes and individual alleles enables the visualization of the presented differences between MG and control cases (Figure 1).

## 4. Discussion

The implication of certain HLA alleles in MG etiopathogenesis has been a topic of interest for the scientific community for the past four decades. However, most of the data concern EOMG and LOMG, defined by different age thresholds and MuSk+MG in populations predominantly of Western European and North American descent and, to a lesser extent, of Chinese and Japanese descent. In this study, we analyzed alleles of five class I and II HLA loci in 40 unrelated Romanian myasthenic patients and investigated the existence of specific allelic associations with MG and its two main serologic subtypes found. We compared allele frequencies in all myasthenic patients and separately patients with RAch+MG and dSNM to controls. The particularity of this research resides in the descent of our patients and also in our approach to separately assess allelic associations with RAch+MG and dSNMG regardless of other clinical or thymic features. Based on the alleles identified to be positively associated with the two serotypes, we were able to discuss the possible etiopathogenic mechanisms by which HLA alleles could contribute to the development of RAch+MG.

The high percentage of women in our group of patients, similar to the one reported in our recent retrospective analysis on a much higher MG cohort of 185 patients, is in concordance with the predominance of EOMG, since this MG subtype is found more frequently in women [1,30].

Overall, in our study the associations between MG and class I HLA alleles outnumbered the ones with class II HLA alleles; this can be partially justified by the predominance of EOMG cases, as this MG subtype is more frequently linked with HLA-A, B and C alleles in both Caucasians and non-Caucasians [7,8,10,31,32,33]. However, this result could suggest that the role of MHC-I restricted T cytotoxic cells may be underestimated in MG development. This theory is supported by the fact that there are several acknowledged mechanisms by which HLA class I alleles can induce autoimmunities, both viral via molecular mimicry, like in the case of certain HLA-A2 alleles that have the ability to recognize certain Influenza A virus epitopes and non-viral such as (1) HLA class I peptides conversion into molecules that will subsequently be presented by HLA class II peptides and (2) inadequate intracellular protein folding, which increases their autoantigenic potential [34,35]. In support of the viral mechanisms by which HLA class I molecules could lead to MG are the results of an experimental study showing that the HLA-B*18:01 allele, with a slightly higher frequency among our myasthenic patients (10% vs. 8.75%), possesses the ability to present an autoantigen homologous to the epitope of Epstein Bar virus (EBV) to the T cytotoxic cells [36]. Since EBV is the only virus that has been isolated from the thymic tissue of myasthenic patients, one can assume that molecular mimicry between this pathogen and self-structures may be involved in MG and might be modulated by HLA-B*18:01 allele [37].

Our results point out not only to previously acknowledged allelic associations with MG but also to some novel ones (Table 6). Since the already known associations confirm the validity of our study, we first focused our attention on them, and afterwards, we discussed the potentially new ones.

### 4.1. Previously Acknowledged Associations Between Myasthenia Gravis and HLA Alleles

In our group of patients, similar to other studies, we found a significant positive association with the HLA-B*08 allele [10,12,13]. The fact that we did not discover associations with any other allele of the ancestral haplotype suggests that B*08 is the singular genetic risk factor for MG from this haplotype. This conclusion is consistent with a GWAS [7] and a case–control study in which whole genome sequencing was performed on 5 EOMG patients and 24 controls homozygous for the AH8.1 haplotype [31].

Similar to other studies which reported them as risk factors for both LOMG in Italian populations [15,16] and MuSK+MG in Caucasian and non-Caucasian populations [18,19,23,38,39,40], DRB1*16 (specifically DRB1*16:01) and DRB1*14 (specifically DRB1*14:54) were positively associated with MG in our analysis. Their associations with MG could be a consequence of the biological role exerted by these HLA class II peptides on exogenous antigen presentation to T helper cells. Moreover, there are several intricate mechanisms that explain association of HLA class II molecules, especially DR/DQ with auto immunities in general, some of which could be extrapolated to MG’s immunopathogeny: (1) exchange of antigen presentation between HLA class II and HLA class I, more specifically, presentation of endogenous antigens with possible repercussions on the triggered immune response; (2) defective MHC restriction of T helper cells, which will allow them to interact with a wider range of antigens; (3) epitope borrowing from another HLA molecule and (4) the presence of polymorphic residues located on the MHC-binding grooves that either could interfere with the selection of functional T regulatory cells or may promote the selection of autoreactive T helper cells [reviewed in 34].

HLA-DQB1*05, which is reported as a risk factor for EOMG [41], LOMG [16] and MuSK+MG [19], was also weakly associated with MG in our study. However, given the known linkage disequilibrium between DQB1*05, DRB1*16 and DRB1*14, along with the higher statistical significance of the associations with the last two alleles and the fact that HLA-DQ*05 frequency among myasthenic patients in our study (42.30%) was lower than in other studies (73% in Italian MuSK+MG patients, 78.3% in Dutch MuSK+MG patients and 42.60% in Italian LOMG patients), it is possible that HLA-DQ*05 is not actually a susceptibility marker for MG in our group of Romanian patients [16,18,19].

DRB1*13 allele was found to be negatively associated with MG in our patients, similar to two studies: one performed on a myasthenic population of Norwegian descent, which pointed DRB1*13:01 as a protective factor for both EOMG and LOMG [17], and another on patients of Serbian descent, which pointed DRB1*13:01 as a protective factor for MuSK+MG [23]. It is important to mention that this allelic association was significant only at the two-digit level, whereas, at the four-digit level, we found no connection between DRB1*13:02—the only HLA-DRB1*13 present in our patients—and MG (Table S1). These different results are most probably due to the comparison of our relatively reduced number of patients with two distinct control groups.

The fact that, in our study, these alleles previously reported as risk or protective factors for certain MG subtypes were positively and negatively, respectively, associated with MG regardless of the clinical and serological heterogeneity of our patients reinforces their potential etiopathogenic role in development or protection, respectively, against the disease.

### 4.2. Novel Associations Between Myasthenia Gravis and HLA Alleles

Even though HLA-B*40 was no longer significantly associated with MG after correction; the fact that its frequency was higher among myasthenic patients compared to the general Romanian population suggested that this allele could be a susceptibility marker. This association, although weak, was a novel positive one. When comparing clinical characteristics between carriers and non-carriers, we found no significant differences regarding gender, bulbar involvement, myasthenic crisis, age at onset or thymic pathology, suggesting that HLA-B*40 could be a potential myasthenic marker. Unexpectedly, myasthenic HLA-B*40 carriers had a significantly higher percentage of autoimmune diseases. This finding suggests that HLA-B*40 either could have a true potential role in MG development or that it is actually a risk factor for other autoimmunities, and therefore, it was falsely positively associated with MG in our study. However, the literature data pointed out that HLA-B*40 is positively associated with autoimmune diseases other than the ones that accompanied MG in our patients, such as late-onset asthma [42] and acquired aplastic anemia [43].

The other alleles (B*47, B*73, A*02:36, B*44:27 and B*57:02) that had a novel positive association with total MG and its two main serotypes were in low numbers (n < 5), and therefore, the extrapolation of the results was limited. We would like to stress that B*47 and B*73 are rare in the entire population of Romania [27]. Furthermore, B*44:27, B*57*02 and A*02:36 have minimal frequencies in Europe (www.allelefrequencies.net, accessed on 6 March 2024). Based on that, our data might be interpreted in two different ways: it could explain their low frequencies in the control groups, thus causing a false-positive association, or their particular presence in the myasthenic group could suggest in fact a potential susceptibility effect. In spite of using the Yates correction for rare events, given the relatively small number of myasthenic patients included, the false-positive scenario may be possible. A potential true positive association between MG and these alleles requires replication in future case–control studies, including a larger number of myasthenic patients. Given the overall rarity of these alleles, a targeted identification followed by retrospective phenotypic comparison of the carriers might be a more conclusive approach.

If indeed the novel allelic associations with MG found in our study are truly positive, these results have potential implications in both experimental and clinical research, with an overall impact on MG management by further clarifying certain immune-mediated mechanisms. Subject to known strong linkage disequilibrium between HLA-A, B and HLA class II alleles, our findings are in concordance with the current acknowledged role of T cytotoxic cells in developing autoimmunity via both non-viral- and viral-mediated mechanisms [34]. Further studies are required in order to test the potential implication of certain viruses in MG development by altering HLA class I expression such as A*02 [35].

The linkage disequilibrium findings might point towards a shared genetic mechanism across autoimmune conditions, thus opening further research directions.

Given the small size of the patients’ group, our results cannot be generalized. However, they offer a fresh Eastern European perspective. Given Romania’s unique genetic background, potential allelic myasthenic population-specific findings can provide insights to MG etiopathogenesis for broader European MG populations.

Regarding the negative associations found between total MG, RAch+MG and especially dSNMG and alleles at the four-digit level, multiple such associations were expected as we have compared alleles, which had zero frequencies in our relatively reduced batch of patients and reduced frequencies in the much numerous control group. Given the small sample size of our study group and therefore the relatively low statistical strength of our study, even though these associations had a statistically significant OR, we cannot firmly acknowledge their protective influence. Therefore, future studies, performed on larger number of samples are recommended.

### 4.3. Considerations Regarding HLA Alleles and the Two Main MG Serotypes (RAch+MG and dSNMG)

The fact that all of the alleles positively associated (B*08, B*47, B*73, A*02:36, B*44:27, B*57:02, DRB1*14:54 and DRB1*16:01) with MG remained significantly associated with RAch+MG, regardless of the patients’ clinical and thymic heterogeneity, suggests that these alleles might play an etiopathogenic role in the tolerance breakdown, which leads to the specific production of autoantibodies. Since the susceptibility alleles were from both HLA classes, both HLA restricted T helper and T cytotoxic cells appear to be involved in MG development. On the grounds that certain HLA alleles code for specific HLA glycoproteins that will influence the specific myasthenic antigen presentation to both T cell populations, either by conformational or electrolytic modifications of the MHC I and II binding grooves, two potential distinct but synergic mechanisms by which HLA alleles may increase susceptibility for MG are suspected [44]. The first mechanism takes place in the peripheral lymphoid organs and involves MHC class II-mediated interactions between B cells and T helper cells: after the naive B cell interacts with the specific myasthenic autoantigen, it will present it to the T helper cell by a MHC II molecule that possesses a particular conformation that provides an increased affinity for the antigen. The T helper cell will then activate the B cells, which, in turn, following the intra-follicular pathway, will transform into B memory cells and plasma cells, the last ones being responsible for autoantibody production [45]. The second mechanism occurs inside the thymus and involves preferential presentation of specific myasthenic antigens by certain MHC I and II grooves to T cells during their positive and negative selection, thus resulting in autoreactive T helper cells escaping the circulation and dysfunction of T regulatory cells (reviewed in [45]). An argument in favor of this latter mechanism is offered by the HLA-DRB1*13 allele, which is suspected to confer a protective role against six autoimmune diseases, including MG, by an optimization of the T cells’ negative selection [46]. In line with this, in our study, the HLA-DRB1*13 allele was significantly negatively associated not only with total MG but also with RAch+MG.

An unexpected result was the positive associations between dSNMG and the HLA-B*47, B*44:27 and DRB1*14:54 alleles, given the fact that no such associations were reported in a study of similar design [47]. The fact that these three alleles were of both HLA classes I and II suggests that both T helper and T cytotoxic cells play a role in the development of dSNMG. The majority of the alleles positively associated with RAch+MG did not maintain this relationship with dSNMG, which suggests that the latter serotype has distinct etiopathogenic mechanisms. However, the common positive associations with B*47, B*44:27 and DRB1*14:54 may be interpreted as common ground, to some extent, for the RAch+MG ‘and dSNMG serotypes. In reality, the extreme heterogeneity of the dSNMG cases makes it difficult to draw definitive conclusions. According to the latest opinion, dSNMG patients defined as seronegative for both RAch and MuSK antibodies can be divided into three subtypes based on the clinical and serological characteristics that may have different possible etiopathogenic mechanisms [48,49]. In two of the subtypes, one with antibodies against RAch (in which patients’ symptoms are similar to EOMG rather than LOMG from a clinical and thymic pathology point of view) and the other with MuSK antibodies, the etiopathogenesis starts in the thymus. It involves either autoantibody production or increased positive selection of autoreactive T helper cells. The third subtype includes a group of patients with particularly severe symptoms that might have a completely distinct pathogenesis [48,49].

It is important to emphasize that all of these associations must be interpreted cautiously given not only the strong linkage disequilibrium within the HLA region but also the fact that the MHC loci are very closely linked and are thus inherited and further transmitted to the offspring as an intact DNA block. Also, one must not forget the possibility of epistasis between epistatic and hypostatic genes [50].

The main strengths of the present study were the high-resolution genotyping of five main HLA class I and II loci performed in a myasthenic population of Eastern European descent, comprehensively segregated in serological MG subtypes. This study pioneers allele associations study in Romanian MG patients and points towards future research directions. However, several limitations must be mentioned. Firstly, the number of myasthenic cases studied was quite limited. This drawback was justified partially by the worldwide rarity of MG and also by the overlap between the studied period and the COVID-19 pandemic years. In addition, a major impediment was the patients’ reluctance to genetic testing. The relatively small size of the study group limited both the overall and the subgroup statistical analysis. As a consequence, the results should be interpreted with caution. Additionally, due to the small number of cases in the MG group, the differences observed between the distribution of HLA-A, HLA-B or HLA-DRB1 haplotypes between MG and control subjects should be interpreted with caution. Secondly, the particular nature of the control groups may be open to further discussions. The design of our study focused on HLA genotyping of myasthenic patients and not on normal individuals. Therefore, following other already published studies, we compared our patients with data from allele frequency databases focusing only on individuals with the same Romanian descent. As the compared variables were of a genetic and not of an environmental nature, there are minimal to no implications for using archived data compared to contemporaneous controls, as long as the two studied groups share a common descent. However, one must acknowledge that the HLA region is highly polymorphic not only in populations of different origin but also between individuals of the same population. In order to augment the internal validity of our research, we included both low- and high-resolution genotyping data. Therefore, given the lack of both levels of resolution genetic data from a single database, we compared our patients’ results with two control groups of the same descent. One must also mention the disproportion among the available data regarding alleles at different resolution levels of the five main HLA loci tested and their frequencies in the general population of Romanian descent. As a consequence, the control group included two subgroups that differed in sample size.

Nonetheless, several future research directions are outlined, alongside specific HLA allelic association with dSNMG, association of MG with other autoimmunities and rare alleles study. All in all, further research on larger number of myasthenic patients is mandatory in order to confirm the allelic associations and to better characterize the differences between the serological subgroups, as well as the linkage disequilibrium data.

## 5. Conclusions

Our HLA analysis of five primary loci in 40 myasthenic patients of Eastern European descent is bringing a novel perspective to a subject that is far from being cleared.

Thus, by finding associations between the HLA-B*08, B*16, B*14 and B*13 alleles and MG, already acknowledged in Western European and North American Caucasian subjects, we have brought further arguments that these MG-related HLA alleles seem to transcend geographical boundaries. If indeed the geographical and perhaps ethnical disparities of the MG-associated HLA genotypes are rather narrow, one can further speculate that, given the common genetic background, the etiopathogenic mechanisms might also be common in MG.

The novel significant positive associations between MG and HLA-A*02:36, B*44:27, B*47, B*57:02 and B*73 that were identified in our study, even though subject to debate due to the rarity of the alleles, offer new research directions for future research. Additionally, future studies are required to confirm if HLA-B*40 is indeed a MG genetic marker. Specifically, the first step consists of larger cohort studies, both national and worldwide, in order to validate the positive associations between MG and these alleles. The next step consists of actively decoding the specific role of these alleles in MG development.

Our results show that the MG cases associating anti-RAch autoantibodies are linked to distinct HLA genes than the double-seronegative patients, suggesting separate etiopathogenic mechanisms for these two entities. However, the existence of three mutual potentially predisposing allelic markers for both RAch+MG and dSNMG suggests, to some extent, a common etiopathogenic ground. Nonetheless, in order to confirm or infirm this theory, future studies, at a larger scale, are required.

## Figures and Tables

**Table 1:** Demographic, clinical and serological characteristics of the 40 myasthenic patients studied.

N = 40 myasthenic patients	
Gender n (%)	
Female	32 (80.00%)
Male	8 (20.00%)
Age at onset mean ± SD (median)	41.45 ± 18.49 (42.5)
MG by age at onset n (%)	
MGj	3 (7.50%)
EOMG	23 (57.50%)
LOMG	14 (35.00%)
MG by muscle groups involved n (%)	
OMG	5 (12.50%)
GMG	35 (87.50%)
Maximum MGFA class	
I	5 (12.50%)
IIA	14 (35.00%)
IIB	11 (27.50%)
IIIB	2 (5.00%)
V	8 (20.00%)
Autoimmune comorbidities n (%)	11 (27.50%)
Thymic assessment n (%)	
Normal CT aspect	22 (55.00%)
Thymoma *	9 (22.50%)
Thymic follicular hyperplasia **	9 (22.50%)
MG serotype n (%)	
RAch+MG	30 (75.00%)
MuSK+MG	1 (2.50%)
dSNMG	9 (22.50%)
Titin and RyR Ab n (%)	
Only titin	1 (2.50%)
Titin and RyR	2 (5.00%)

SD, standard deviation; MG, myasthenia gravis; MGj, juvenile MG; EOMG, early-onset MG; LOMG, late-onset MG; MGFA, Myasthenia Gravis Foundation of America classification; CT, computer tomography; * histological confirmation in 8 out of 9 cases; ** histological confirmation in 7 out of 9 cases; RAch+MG, MG with acetylcholine receptor; MuSK+MG, MG with muscle-specific receptor tyrosine kinase; dSNMG, double-seronegative MG; Ab, antibody; RyR, ryanodine receptor.

**Table 2:** HLA class I and II (A, B, C, DRB1 and DQB1) alleles (two-digit level) frequency analysis in the total MG group, MG subgroups according to autoantibody profile vs. the healthy controls group.

HLA-A	MG 100% (80)	dSNMG 100% (18)	RAch+MG 100% (60)	Control Group 100% (11,516)
A*01	16.25% (13)	11.11% (2)	15.00% (9)	12.00%
A*02	35.00% (28)	44.44% (8)	33.33% (20)	26.00%
A*03	7.50% (6)	5.55% (1)	8.33% (5)	9.00%
A*11	2.50% (2)	0.00% (0)	3.33% (2)	8.00%
A*23	1.25% (1)	0.00% (0)	1.67% (1)	3.00%
A*24	15.00% (12)	22.22% (4)	13.33% (8)	12.00%
A*25	3.75% (3)	5.55% (1)	3.33% (2)	3.00%
A*26	5.00% (4)	0.00% (0)	6.67% (4)	5.00%
A*29	1.25% (1)	0.00% (0)	1.67% (1)	2.00%
A*30	1.25% (1)	5.55% (1)	0.00% (0)	2.00%
A*31	1.25% (1)	0.00% (0)	1.67% (1)	2.00%
A*32	6.25% (5)	0.00% (0)	8.33% (5)	4.00%
A*33	2.50% (2)	5.55% (1)	1.67% (1)	3.00%
A*36	0.00% (0)	0.00% (0)	0.00% (0)	4.00%
A*68	1.25% (1)	0.00% (0)	1.67% (1)	3.00%
**HLA-B**	**MG** **100% (80)**	**dSNMG** **100% (18)**	**RAch+MG** **100% (60)**	**Control Group** **100% (12,020)**
B*07	3.75% (3)	0.00% (0)	5.00% (3)	5.00%
B*08	15.00% (12) **^a^**	11.11% (2)	15.00% (9) **^i^**	6.00%
B*13	3.75% (3)	5.55% (1)	3.33% (2)	3.00%
B*14	0.00% (0)	0.00% (0)	0.00% (0)	2.00%
B*15	2.50% (2)	0.00% (0)	3.33% (2)	3.00%
B*18	11.25% (9)	16.67% (3)	10.00% (6)	10.00%
B*27	5.00% (4)	5.55% (1)	5.00% (3)	4.00%
B*35	13.75% (11)	11.11% (2)	15.00% (9)	14.00%
B*38	3.75% (3)	5.55% (1)	3.33% (2)	3.00%
B*39	2.50% (2)	0.00% (0)	3.33% (2)	3.00%
B*40	10.00% (8) **^b^**	11.11% (2)	10.00% (6)	5.00%
B*41	0.00% (0)	0.00% (0)	0.00% (0)	2.00%
B*44	8.75% (7)	5.55% (1)	10.00% (6)	8.00%
B*47	2.50% (2) **^c^**	5.55% (1) **^h^**	1.67% (1) **^j^**	0.00%
B*49	0.00% (0)	0.00% (0)	0.00% (0)	2.00%
B*50	0.00% (0)	0.00% (0)	0.00% (0)	2.00%
B*51	10.00% (8)	11.11% (2)	8.33% (5)	9.00%
B*52	1.25% (1)	0.00% (0)	1.67% (1)	4.00%
B*53	0.00% (0)	0.00% (0)	0.00% (0)	2.00%
B*55	1.25% (1)	5.55% (1)	0.00% (0)	2.00%
B*56	0.00% (0)	0.00% (0)	0.00% (0)	1.00%
B*57	2.50% (2)	0.00% (0)	3.33% (2)	1.00%
B*58	1.25% (1)	5.55% (1)	0.00% (0)	2.00%
B*78	0.00% (0)	0.00% (0)	0.00% (0)	3.00%
B*73	1.25% (1) **^d^**	0.00% (0)	1.67% (1) **^k^**	0.00%
**HLA-C**	**MG** **100% (80)**	**dSNMG** **100% (18)**	**RAch+MG** **100% (60)**	**Control Group 100% (3224)**
C*01	5.00% (4)	16.67% (3)	1.67% (1)	5.00%
C*02	10.00% (8)	11.11% (2)	10.00% (6)	7.00%
C*03	7.50% (6)	5.55% (1)	8.33% (5)	7.00%
C*04	10.00% (8)	5.55% (1)	11.67% (7)	15.00%
C*05	1.25% (1)	0.00% (0)	1.67% (1)	3.00%
C*06	11.25% (9)	11.11% (2)	11.67% (7)	9.00%
C*07	31.25% (25)	8.33% (5)	31.67% (19)	26.00%
C*08	0.00% (0)	0.00% (0)	0.00% (0)	3.00%
C*12	12.50% (10)	11.11% (2)	11.67% (7)	13.00%
C*14	2.50% (2)	5.55% (1)	1.67% (1)	3.00%
C*15	6.25% (5)	5.55% (1)	6.67% (4)	5.00%
C*16	2.50% (2)	0.00% (0)	3.33% (2)	3.00%
C*17	0.00% (0)	0.00% (0)	0.00% (0)	1.00%
**HLA-DRB1**	**MG** **100% (80)**	**dSNMG** **100% (18)**	**RAch+MG** **100% (60)**	**Control Group 100% (13,872)**
DRB1*01	11.25% (9)	11.11% (2)	11.67% (7)	8.00%
DRB1*03	10.00% (8)	5.55% (1)	11.67% (7)	11.00%
DRB1*04	8.75% (7)	5.55% (1)	10.00% (6)	9.00%
DRB1*07	7.50% (6)	11.11% (2)	5.00% (3)	7.00%
DRB1*08	0.00% (0)	0.00% (0)	0.00% (0)	2.00%
DRB1*10	2.50% (2)	5.55% (1)	1.67% (1)	1.00%
DRB1*11	17.50% (14)	22.22% (4)	16.67% (10)	19.00%
DRB1*12	1.25% (1)	0.00% (0)	1.67% (1)	2.00%
DRB1*13	1.25% (1) **^e^**	5.55% (1)	0.00% (0) **^l^**	15.00%
DRB1*14	10.00% (8)	16.67% (3)	6.67% (4)	7.00%
DRB1*15	12.50% (10)	5.55% (1)	15.00% (9)	9.00%
DRB1*16	17.50% (14) **^f^**	11.11% (2)	20.00% (12) **^m^**	10.00%
**HLA-DQB1**	**MG** **100% (78)**	**dSNMG** **100% (18)**	**RAch+MG** **100% (58)**	**Control Group 100% (3328 × 2 = 6656)**
DQB1*02	14.10% (11)	16.67% (3)	13.80% (8)	21.00%
DQB1*03	29.48% (23)	8.33% (5)	29.31% (17)	29.00%
DQB1*04	0.00% (0)	0.00% (0)	0.00% (0)	2.00%
DQB1*05	42.30% (33) **^g^**	44.44% (8)	41.37% (24)	30.00%
DQB1*06	14.10% (11)	11.11% (2)	15.51% (9)	18.00%

MG, myasthenia gravis; dSNMG, double-seronegative MG; RAch+MG, MG with acetylcholine receptor; OR, odds ratio; CI, confidence interval; *p*c, *p*-values corrected using the Benjamini–Hochberg method. ^a^ MG vs. controls: *p* = 0.0008, *p*c = 0.00666, OR = 2.7484, 95% CI = 1.4810–5.1004, Z = 3.205, *p* = 0.0014. ^b^ MG vs. controls: *p* = 0.0415, *p*c = 0.25937, OR = 2.1111, 95% CI = 1.0122–4.4029, Z = 1.992, *p* = 0.0463. ^c^ MG vs. controls: *p* < 0.00001, *p*c = 0.000125, OR = 765.6369, 95% CI = 36.4590–16,078.3238, Z = 4.275, *p* < 0.0001. ^d^ MG vs. controls: *p* < 0.00001, *p*c = 0.000125, OR = 453.6038, 95% CI = 18.3379–11,220.3047, Z = 3.737, *p* = 0.0002. ^e^ MG vs. controls: *p* = 0.001, *p*c = 0.012, OR = 0.0717, 95% CI = 0.0100–0.5158, Z = 2.618, *p* = 0.0089. ^f^ MG vs. controls: *p* = 0.026, *p*c = 0.156, OR = 1.9094, 95% CI = 1.0697–3.4081, Z = 2.188, *p* = 0.0287. ^g^ MG vs. controls: *p* = 0.029, *p*c = 0.145, OR = 1.6381, 95% CI = 1.0463–2.5644, Z = 2.158, *p* = 0.0309. ^h^ dSNMG vs. controls: *p* < 0.00001, *p*c = 0.00025, OR = 2060.6571, 95% CI = 81.1219–52,344.7694, Z = 4.624, *p* < 0.00001. ^i^ RAch+MG vs. controls: *p* = 0.0035, *p*c = 0.02916, OR = 2.7655, 95% CI = 1.3561–5.6398, Z = 2.798, *p* = 0.0051. ^j^ RAch+MG vs. controls: *p* < 0.00001, *p*c = 0.000125, OR = 606.0756, 95% CI = 24.4399–15,029.8190, Z = 3.911, *p* = 0.0001. ^k^ RAch+MG vs. controls: *p* < 0.00001, *p*c = 0.000125, OR = 606.0756, 95% CI = 24.4399–15,029.8190, Z = 3.911, *p* = 0.0001. ^l^ RAch+MG vs. controls: *p* = 0.0021, *p*c = 0.0252, OR = 0.0468, 95% CI = 0.0029–0.7574, Z = 2.156, *p* = 0.0311. ^m^ RAch+MG vs. controls: *p* = 0.018, *p*c = 0.1079, OR = 2.2504, 95% CI = 1.1925–4.2465, Z = 2.503, *p* = 0.0123.

**Table 3:** Comparative analysis of demographic, clinical and thymic characteristics of B*40 carriers and non-carriers (chi-square test).

Characteristics	B*40 Carriers (8)	B*40 Non-Carriers (32)	*p*
Females	8 (100%)	24 (75.00%)	0.28
EOMG	6 (75.00%)	17 (53.12%)	0.47
LOMG	1 (12.50%)	13 (40.62%)	0.28
MGj	1 (12.50%)	2 (6.25%)	0.88
Maximum MGFA			
I	0	5 (15.62%)	0.55
IIA	3 (37.50%)	11 (34.37%)	0.80
V	2 (25%)	6 (18.75%)	0.92
Bulbar involvement (IIB+IIIB)	3 (37.50%)	10 (31.25%)	0.93
Autoimmune comorbidities	5 (62.50%)	6 (18.75%)	0.042
Thymoma	3 (37.50%)	6 (18.75%)	0.51
Thymic follicular hyperplasia	1 (12.50%)	8 (25.00%)	0.78

MG, myasthenia gravis; MGj, juvenile MG; EOMG, early-onset MG; LOMG, late-onset MG; MGFA, Myasthenia Gravis Foundation of America classification.

**Table 4:** HLA class I and II (A, B, C, DRB1) alleles (four-digit level) significantly associated (chi-square test) after correction with RAch+MG and the quantification of these associations.

Allele	RAch+MG 100% (60)	Control Group 100% (2468)	*p*	*p*c	OR	95% CI	Z	*p*
A*02:07	0.00% (0)	0.04%	0.0018	0.0078	13.5950	0.5482–337.1467	1.593	0.1111
A*02:08	0.00% (0)	0.04%	0.0018	0.0078	13.5950	0.5482–337.1467	1.593	0.1111
A*02:27	0.00% (0)	0.04%	0.0018	0.0078	13.5950	0.5482–337.1467	1.593	0.1111
A*02:36	**1.67% (1)**	**0.00%**	**0.0018**	**0.0078**	**124.4622**	**5.0180** **–3087.0807**	**2.945**	**0.0032**
A*03:97	0.00% (0)	0.04%	0.0018	0.0078	13.5950	0.5482–337.1467	1.593	0.1111
A*24:18	0.00% (0)	0.04%	0.0018	0.0078	13.5950	0.5482–337.1467	1.593	0.1111
A*68:18N	0.00% (0)	0.04%	0.0018	0.0078	13.5950	0.5482–337.1467	1.593	0.1111
A*74:01	0.00% (0)	0.04%	0.0018	0.0078	13.5950	0.5482–337.1467	1.593	0.1111
A*80:01	0.00% (0)	0.04%	0.0018	0.0078	13.5950	0.5482–337.1467	1.593	0.1111
B*15:09	0.00% (0)	0.04%	0.0018	0.01047	13.5950	0.5482–337.1467	1.593	0.1111
B*15:39	0.00% (0)	0.04%	0.0018	0.01047	13.5950	0.5482–337.1467	1.593	0.1111
B*15:73	0.00% (0)	0.04%	0.0018	0.01047	13.5950	0.5482–337.1467	1.593	0.1111
B*35:14	0.00% (0)	0.04%	0.0018	0.01047	13.5950	0.5482–337.1467	1.593	0.1111
B*39:10	0.00% (0)	0.04%	0.0018	0.01047	13.5950	0.5482–337.1467	1.593	0.1111
B*39:31	0.00% (0)	0.04%	0.0018	0.01047	13.5950	0.5482–337.1467	1.593	0.1111
B*44:04	0.00% (0)	0.04%	0.0018	0.01047	13.5950	0.5482–337.1467	1.593	0.1111
B*44:27	**1.67% (1)**	**0.00%**	**<0.00001**	**0.00032**	**210.9829**	**10.0172** **–4443.7135**	**3.442**	**0.0006**
B*51:02	0.00% (0)	0.05%	0.0018	0.01047	13.5950	0.5482–337.1467	1.593	0.1111
B*53:01	0.00% (0)	0.04%	0.0018	0.01047	13.5950	0.5482–337.1467	1.593	0.1111
B*57:02	**3.33% (2)**	**0.08%**	**<0.00001**	**0.00032**	**42.5172**	**5.8867–307.0860**	**3.717**	**0.0002**
C*08:01	0.00% (0)	0.04%	0.0018	0.0126	13.5950	0.5482–337.1467	1.593	0.1111
C*12:12	0.00% (0)	0.04%	0.0018	0.0126	13.5950	0.5482–337.1467	1.593	0.1111
C*15:06	0.00% (0)	0.04%	0.0018	0.0126	13.5950	0.5482–337.1467	1.593	0.1111
C*18:01	0.00% (0)	0.04%	0.0018	0.0126	13.5950	0.5482–337.1467	1.593	0.1111
DRB1*01:11	0.00% (0)	0.04%	0.0018	0.0096	13.5950	0.5482–337.1467	1.593	0.1111
DRB1*11:11	0.00% (0)	0.04%	0.0018	0.0096	13.5950	0.5482–337.1467	1.593	0.1111
DRB1*11:33	0.00% (0)	0.04%	0.0018	0.0096	13.5950	0.5482–337.1467	1.593	0.1111
DRB1*11:39	0.00% (0)	0.04%	0.0018	0.0096	13.5950	0.5482–337.1467	1.593	0.1111
DRB1*11:43	0.00% (0)	0.04%	0.0018	0.0096	13.5950	0.5482–337.1467	1.593	0.1111
DRB1*13:22	0.00% (0)	0.04%	0.0018	0.0096	13.5950	0.5482–337.1467	1.593	0.1111
DRB1*14:06	0.00% (0)	0.04%	0.0018	0.0096	13.5950	0.5482–337.1467	1.593	0.1111
DRB1*14:54	**3.33% (2)**	**0.00%**	**<0.00001**	**0.00048**	**210.9829**	**10.0172–4443.7135**	**3.442**	**0.0006**
DRB1*16:01	**18.33%**	**6.44%**	**0.0003**	**0.0072**	**3.2600**	**1.6625–6.3926**	**3.440**	**0.0006**

MG, myasthenia gravis; RAch+MG, MG with acetylcholine receptor; OR, odds ratio; CI, confidence interval; *p*c, *p*-values corrected using Benjamini–Hochberg method; **bold**, significant OR.

**Table 5:** HLA-A, HLA-B and HLA-DRB1 haplotypes in MG and control cases.

HLA-A Haplotype	MG (%)	Control (%)	HLA-B Haplotype	MG (%)	Control (%)	HLA-DRB1 Haplotype	MG (%)	Control (%)
A*01-A*01	2.50	2.41	B*07-B*35	**5.00**	1.62	DRB1*01-DRB1*03	2.50	3.73
A*01-A*02	**10.00**	**10.46**	B*07-B*44	2.50	1.35	DRB1*01-DRB1*07	**5.00**	0.75
A*01-A*03	**7.50**	1.61	B*08-B*15	2.50	0.27	DRB1*01-DRB1*11	**5.00**	2.24
A*01-A*24	**5.00**	4.29	B*08-B*18	**5.00**	2.70	DRB1*01-DRB1*14	**5.00**	0.75
A*01-A*32	**5.00**	0.80	B*08-B*35	2.50	1.89	DRB1*01-DRB1*15	2.50	0.75
A*02-A*02	**12.50**	**6.97**	B*08-B*38	2.50	0.27	DRB1*01-DRB1*16	2.50	0.75
A*02-A*03	2.50	**6.43**	B*08-B*39	2.50	0.81	DRB1*03-DRB1*11	2.50	2.99
A*02-A*11	2.50	4.02	B*08-B*40	2.50	1.08	DRB1*03-DRB1*14	**5.00**	2.24
A*02-A*24	**15.00**	**6.17**	B*08-B*44	**5.00**	1.08	DRB1*03-DRB1*15	2.50	2.99
A*02-A*25	2.50	1.34	B*08-B*51	2.50	1.35	DRB1*03-DRB1*16	**10.00**	2.24
A*02-A*26	**5.00**	2.95	B*08-B*55	2.50	0.00	DRB1*04-DRB1*13	2.50	2.99
A*02-A*29	2.50	0.00	B*08-B*73	2.50	0.00	DRB1*04-DRB1*15	**7.50**	1.49
A*02-A*30	2.50	1.07	B*13-B*27	2.50	0.00	DRB1*04-DRB1*16	**7.50**	1.49
A*02-A*32	2.50	2.14	B*13-B*35	2.50	0.81	DRB1*07-DRB1*11	**5.00**	**5.22**
A*03-A*32	2.50	0.54	B*13-B*51	2.50	1.35	DRB1*07-DRB1*14	2.50	0.75
A*03-A*33	2.50	0.00	B*15-B*18	2.50	1.89	DRB1*07-DRB1*15	2.50	0.75
A*11-A*24	2.50	0.80	B*18-B*38	2.50	1.08	DRB1*10-DRB1*14	2.50	0.00
A*23-A*68	2.50	0.00	B*18-B*39	2.50	0.27	DRB1*11-DRB1*11	2.50	2.99
A*24-A*24	2.50	1.88	B*18-B*40	5.00	1.35	DRB1*11-DRB1*12	2.50	0.75
A*24-A*25	2.50	1.61	B*18-B*44	2.50	2.16	DRB1*11-DRB1*15	**5.00**	4.48
A*25-A*33	2.50	0.00	B*18-B*51	2.50	2.70	DRB1*11-DRB1*16	**10.00**	**5.22**
A*26-A*26	2.50	0.00	B*27-B*35	2.50	0.81	DRB1*14-DRB1*15	2.50	0.00
A*31-A*32	2.50	0.80	B*27-B*51	2.50	0.54	DRB1*14-DRB1*16	2.50	2.99
			B*27-B*57	2.50	0.27	DRB1*15-DRB1*16	2.50	2.24
			B*35-B*40	**5.00**	1.62			
			B*35-B*44	**5.00**	2.16			
			B*35-B*51	2.50	2.16			
			B*35-B*52	2.50	1.35			
			B*38-B*57	2.50	0.27			
			B*40-B*47	**5.00**	0.00			
			B*40-B*51	2.50	0.81			
			B*44-B*51	2.50	1.35			
			B*51-B*58	2.50	0.27			
**n**	40	373	**n**	40	371	**n**	40	134
*p* < 0.0001			*p* < 0.0001			*p* < 0.0001		
HLA-A haplotypes			HLA-B haplotypes			HLA-DRB1 haplotypes		
HW test	MG	Control	HW test	MG	Control	HW test	MG	Control
*p* (chi-square)	<0.0001	0.0027	*p* (chi-square)	<0.0001	<0.0001	*p* (chi-square)	0.0250	0.0831

MG, myasthenia gravis; n, number of individuals; HW, Hardy–Weinberg equilibrium test; bold, values ≥ 5%.

**Table 6:** Positive associations (after Benjamini–Hochberg correction and with a significant OR) between MG and HLA alleles in our study vs. literature data.

HLA Alelle	*p*	*p*c	Previously Acknowledged by
A*02:36	0.007	0.0312	none
B*08	0.0008	0.00666	7, 10, 12, 13, 31
B*44:27	<0.00001	0.00064	none
B*47	<0.00001	0.000125	none
B*57:02	0.0001	0.0032	none
B*73	<0.00001	0.000125	none
DRB1*14:54	<0.00001	0.00048	DRB1*14—15, 18, 38, 39, 40
DRB1*16:01	0.0006	0.0144	DRB1*16—16, 18, 19, 23, 38, 39

*p*c, *p*-values corrected using Benjamini–Hochberg method; **bold**, significant positive association also with RAch+MG; *italics*, significant positive association also with dSNMG.

## Data Availability

The data presented in this study are available on request from the corresponding author. The data are not publicly available due to privacy restrictions.

## Supplementary Materials

The following supporting information can be downloaded at https://www.mdpi.com/article/10.3390/neurolint16060130/s1: Table S1: Associations between HLA class I and II alleles (genotyped at the four-digit level) and MG; Table S2: HLA class I and II (A, B, C and DRB1) alleles (four-digit level) significantly associated (chi-square test) after correction with dSNMG and the quantification of these associations; Table S3: Linkage disequilibrium in the MG group and the control group.
