# Supplementary material for: HLA Class I and II Alleles in Anti-Acetylcholine Receptor Antibodies Positive and Double-Seronegative Myasthenia Gravis Patients of Romanian Descent
Source: Neurol Int. 2024 Dec 10;16(6):1819–36. doi: 10.3390/neurolint16060130 (PMC11676662; doi:10.3390/neurolint16060130)
Supplement: Supplementary file 1 [file neurolint-16-00130-s001.zip › neurolint-3218265-supplementary.pdf]

Supplementary Material

Table S1. Associations between HLA class I and II alleles (genotyped at the four-digit level) and MG

HLA-A	MG 100% (80)	Control group 100% (2468)	*p*	*p*c	OR	95%IC	Z	*p*
A*01:01	16.25% (13)	14.57%	0.68	0.9471				
A*02:01	33.75% (27)	27.63%	0.23	0.5276				
A*02:02	0% (0)	0.17%	0.38	0.6736				
A*02:05	0% (0)	0.73%	0.93	0.975				
A*02:06	0% (0)	0.12%	0.18	0.5014				
A*02:07	0% (0)	0.04%	0.0072	0.0312				
A*02:08	0% (0)	0.04%	0.0072	0.0312	10.2174	0.4130 – 252.7616	1.420	0.1557
A*02:11	0% (0)	0.16%	0.28	0.5276				
A*02:17	0% (0)	0.32%	0.61	0.915				
A*02:27	0% (0)	0.04%	0.0072	0.0312	10.2174	0.4130 - 252.7616	1.420	0.1557
A*02:30	0% (0)	0,08%	0,076	0,2964				
**A*02:36**	1.25% (1)	0%	**0.007**	**0.0312**	93.1509	3.7651 – 2304.6180	2.770	**0.0056**
A*03:01	6.25% (5)	11.75%	0.13	0.4609				
A*03:02	1.25% (1)	0.61%	0.99	0.99				

A*03:97	0% (0)	0.04%	0.0072	0.0312	10.2174	0.4130 – 252.7616	1.420	0.1557
A*11:01	2.50% (2)	5.84%	0.31	0.6045				
A*23:01	1.25% (1)	1.99%	0.95	0.975				
A*24:02	15.00% (12)	10.79%	0.23	0.5276				
A*24:03	0% (0)	0.41%	0.74	0.975				
A*24:18	0% (0)	0.04%	0.0072	0.0312	10.2174	0.4130 – 252.7616	1.420	0.1557
A*25:01	3.75% (3)	2.96%	0.94	0.975				
A*26:01	5.00% (4)	4.17%	0.94	0.975				
A*29:01	0% (0)	0.53%	0.88	0.975				
A*29:02	1.25% (1)	1.70%	0.89	0.975				
A*30:01	0% (0)	1.66%	0.48	0.8139				
A*30:02	1.25% (1)	0.77%	0.89	0.975				
A*30:04	0% (0)	0.12%	0.18	0.5014				
A*31:01	1.25% (1)	2.88%	0.60	0.915				
A*32:01	6.25% (5)	3.65%	0.36	0.6685				
A*33:01	0% (0)	1.30%	0.61	0.915				
A*33:03	2.50% (2)	0.69%	0.23	0.5276				
A*34:02	0% (0)	0.16%	0.28	0.5276				
A*66:01	0% (0)	0.57%	0.93	0.975				
A*68:01	1.25% (1)	2.71%	0.65	0.9388				

A*68:02	0% (0)	0.45%	0.79	0.975				
A*68:18N	0% (0)	0.04%	0.0072	0.0312	10.2174	0.4130 – 252.7616	1.420	0.1557
A*74:01	0% (0)	0.04%	0.0072	0.0312	10.2174	0.4130 – 252.7616	1.420	0.1557
A*74:03	0% (0)	0.12%	0.18	0.5014				
A*80:01	0% (0)	0.04%	0.0072	0.0312	10.2174	0.4130 – 252.7616	1.420	0.1557
HLA-B	MG<br>100% (80)	Control group 100%<br>(2468)						
B*07:02	3.75% (3)	7.46%	0.30	0.6012				
B*07:04	0% (0)	0.08%	0.076	0.2432				
B*07:05	0% (0)	0.53%	0.88	0.9752				
B*08:01	15.00% (12)	9.48%	0.10	0.3047				
B*13:02	3.75% (3)	3.57%	0.83	0.9752				
B*14:01	0% (0)	0.16 %	0.28	0.6012				
B*14:02	0% (0)	2.39 %	0.31	0.6012				
B*15:01	1.25% (1)	5.07 %	0.20	0.512				
B*15:03	0% (0)	0.08 %	0.076	0.2432				
B*15:08	0% (0)	0.08 %	0.076	0.2432				
B*15:09	0% (0)	0.04 %	0.0072	0.04189	10.2174	0.4130 – 252.7616	1.420	0.1557
B*15:17	1.25% (1)	0.53 %	0.93	0.9752				
B*15:18	0 %(0)	0.16 %	0.28	0.6012				

B*15:39	0% (0)	0.04 %	0.0072	0.04189	10.2174	0.4130 – 252.7616	1.420	0.1557
B*15:73	0% (0)	0.04 5	0.0072	0.04189	10.2174	0.4130 – 252.7616	1.420	0.1557
B*18:01	10.00% (8)	8.75%	0.70	0.9665				
B*18:03	0% (0)	0.16 %	0.28	0.6012				
B*18:04	0% (0)	0.08 %	0.076	0.2432				
B*18:05	1.25% (1)	0.32 %	0.68	0.9665				
B*27:02	2.50% (2)	1.54 %	0.82	0.9752				
B*27:05	2.50% (2)	3.04 %	0.96	0.9752				
B*27:07	0% (0)	0.08 %	0.076	0.2432				
B*35:01	5.00% (4)	5.71 %	0.98	0.98				
B*35:02	3.75% (3)	1.82 %	0.41	0.7288				
B*35:03	3.75% (3)	3.93 %	0.83	0.9752				
B*35:08	1.25% (1)	1.05 %	0.70	0.9665				
B*35:14	0% (0)	0.04 %	0.0072	0.04189	10.2174	0.4130 – 252.7616	1.420	0.1557
B*37:01	0% (0)	1.26 %	0.62	0.9665				
B*38:01	3.75% (3)	3.49 %	0.86	0.9752				
B*39:01	2.50% (2)	1.78 %	0.96	0.9752				
B*39:06	0% (0)	0.49 %	0.84	0.9752				
B*39:10	0% (0)	0.04 %	0.0072	0.04189	10.2174	0.4130 – 252.7616	1.420	0.1557
B*39:31	0% (0)	0.04 %	0.0072	0.04189	10.2174	0.4130 – 252.7616	1.420	0.1557

B*40:01	2.50% (2)	2.48 %	0.73	0,9665				
B*40:02	3.75% (3)	1.58 %	0.29	0.6012				
B*40:06	3.75% (3)	0.73%	0.02	0.09846	5.3030	1.5298 - 18.3830	2.630	0.0085
B*41:01	0% (0)	0.73%	0.93	0.9752				
B*41:02	0% (0)	0.93%	0.79	0.9752				
B*44:02	2.50% (2)	5.79%	0.31	0.6012				
B*44:03	2.50% (2)	3.32%	0.93	0.9752				
B*44:04	0% (0)	0.04%	0.0072	0.04189	10.2174	0.4130 – 252.7616	1.420	0.1557
B*44:05	1.25% (1)	1.14%	0.66	0.9665				
**B*44:27**	2.50% (2)	0%	**<0.00001**	**0.00064**	157.2293	7.4856 - 3302.4752	3.256	**0.0011**
B*45:01	0% (0)	0.41%	0.74	0.9665				
B*46:01	0% (0)	0.08%	0.076	0.2432				
B*47:01	2.50% (2)	0.28%	0.02	0.09846	9.0147	1.8428 - 44.0988	2.715	0.0066
B*48:01	0% (0)	0.16%	0.28	0.6012				
B*49:01	0% (0)	1.62%	0.49	0.8475				
B*50:01	0% (0)	1.46%	0.54	0.8861				
B*51:01	8.75% (7)	7.42%	0.65	0.9665				
B*51:02	0% (0)	0.05%	0.0072	0.04189	10.2174	0.4130 – 252.7616	1.420	0.1557
B*51:05	0% (0)	0.08%	0.076	0.2432				
B*51:07	0% (0)	0.13%	0.18	0.48				

B*51:08	1.25% (1)	0.16%	0.38	0.6948				
B*51:65	0% (0)	0.12%	0.18	0.48				
B*52:01	1.25% (1)	1.38%	0.70	0.9665				
B*53:01	0% (0)	0.04%	0.0072	0.04189	10.2174	0.4130 – 252.7616	1.420	0.1557
B*55:01	1.25% (1)	2.07%	0.92	0.9752				
B*56:01	0% (0)	0.73%	0.93	0.9752				
B*57:01	0% (0)	2.31%	0.32	0.6023				
**B*57:02**	2.50% (2)	0.08%	**0.0001**	**0.0032**	31.6154	4.3961 - 227.3664	3.431	**0.0006**
B*57:03	0% (0)	0.24%	0.54	0.8861				
B*58:01	1.25% (1)	0.97%	0.74	0.9665				
B*73:01	1.25% (1)	0.08%	0.18	0.48				
HLA-C	MG<br><br>100% (80)	Control group 100%<br><br>(2468)						
C*01:02	5.00% (4)	3.97%	0.86	0.99				
C*02:02	10.00% (8)	6.77%	0.26	0.7466				
C*03:02	0% (0)	0.45%	0.79	0.99				
C*03:03	3.75% (3)	4.54%	0.95	0.99				
C*03:04	3.75% (3)	4.13%	0.91	0.99				
C*04:01	10.00% (8)	13.86%	0.32	0.7466				

C*05:01	1.25% (1)	5.51%	0.16	0.63				
C*06:02	11.25% (9)	9.05%	0.50	0.91				
C*06:17	0% (0)	0.19%	0.38	0.8184				
C*07:01	21.25% (17)	17.03%	0.32	0.7466				
C*07:02	6.25% (5)	8.79%	0.43	0.86				
C*07:04	3.75% (3)	1.26%	0.16	0.63				
C*08:01	0% (0)	0.04%	0.0072	0.0504	10.2174	0.4130 – 252.7616	1.420	0.1557
C*08:02	0% (0)	2.55%	0.28	0.7466				
C*08:03	0% (0)	0.12%	0.18	0.63				
C*12:02	2.50% (2)	1.30%	0.67	0.99				
C*12:03	10.00% (8)	9.87%	0.97	0.99				
C*12:12	0% (0)	0.04%	0.0072	0.0504	10.2174	0.4130 – 252.7616	1.420	0.1557
C*14:02	2.50% (2)	1.54%	0.82	0.99				
C*15:02	5.00% (4)	3.98%	0.86	0.99				
C*15:04	0% (0)	0.12%	0.18	0.63				
C*15:05	1.25% (1)	0.61%	0.99	0.99				
C*15:06	0% (0)	0.04%	0.0072	0.0504	10.2174	0.4130 – 252.7616	1.420	0.1557
C*16:01	1.25% (1)	1.54%	0.80	0.99				
C*16:02	1.25% (1)	0.65%	0.96	0.99				

C*16:04	0% (0)	0.32%	0.61	0.99				
C*17:01	0% (0)	1.54%	0.52	0.91				
C*18:01	0% (0)	0.04%	0.0072	0.0504	10.2174	0.4130 – 252.7616	1.420	0.1557
HLA-DRB1	MG 100% (80)	Control group 100% (2468)						
DRB1*01:01	11.25% (9)	8.55%	0.40	0.640				
DRB1*01:02	0% (0)	1.26%	0.62	0.7258				
DRB1*01:03	0% (0)	0.12%	0.18	0.3927				
DRB1*01:11	0% (0)	0.04%	0.0072	0.0384	10.2174	0.4130 – 252.7616	1.420	0.1557
DRB1*03:01	10.00% (8)	11.91%	0.60	0.7258				
DRB1*04:01	0% (0)	4.30%	0.11	0.33				
DRB1*04:02	1.25% (1)	2.19%	0.86	0.8987				
DRB1*04:03	2.50% (2)	1.22%	0.61	0.7258				
DRB1*04:04	3.75% (3)	2.27%	0.62	0.7258				
DRB1*04:05	0% (0)	0.53%	0.88	0.8987				
DRB1*04:07	1.25% (1)	0.45%	0.84	0.8987				
DRB1*04:08	0% (0)	0.16%	0.28	0.56				
DRB1*04:10	0% (0)	0.08%	0.076	0.2432				
DRB1*07:01	7.50% (6)	11.10%	0.31	0.5907				

DRB1*08:01	0% (0)	2.23%	0.34	0.60				
DRB1*08:02	0% (0)	0.08%	0.076	0.2432				
DRB1*08:03	0% (0)	0.12%	0.18	0.3927				
DRB1*08:04	0% (0)	0.12%	0.18	0.3927				
DRB1*08:17	0% (0)	0.08%	0.076					
DRB1*09:01	0% (0)	0.53%	0.88	0.8987				
DRB1*10:01	2.50% (2)	1.22%	0.61	0.7258				
DRB1*11:01	6.25% (5)	7.29%	0.72	0.8228				
DRB1*11:02	0% (0)	0.20%	0.38	0.6289				
DRB1*11:03	0% (0)	1.58%	0.50	0.7058				
DRB1*11:04	11.25% (9)	7.94%	0.28	0.56				
DRB1*11:08	0% (0)	0.08%	0.076	0.2432				
DRB1*11:11	0% (0)	0.04%	0.0072	0.0384	10.2174	0.4130 – 252.7616	1.420	0.1557
DRB1*11:15	0% (0)	0.12%	0.18	0.3927				
DRB1*11:33	0% (0)	0.04%	0.0072	0.0384	10.2174	0.4130 – 252.7616	1.420	0.1557
DRB1*11:39	0% (0)	0.04%	0.0072	0.0384	10.2174	0.4130 – 252.7616	1.420	0.1557
DRB1*11:43	0% (0)	0.04%	0.0072	0.0384	10.2174	0.4130 – 252.7616	1.420	0.1557
DRB1*12:01	0% (0)	1.74%	0.47	0.6836				
DRB1*12:02	1.25% (1)	0.08%	0.18	0.3927				
DRB1*13:01	0% (0)	7.78%	0.017	0.0816	0.0735	0.0045 – 1.1891	1.838	0.0661

DRB1*13:02	1.25% (1)	2.31%	0.81	0.8987				
DRB1*13:03	0% (0)	1.50%	0.53	0.7066				
DRB1*13:05	0% (0)	0.12%	0.18	0.3927				
DRB1*13:22	0% (0)	0.04%	0.0072	0.0384	10.2174	0.4130 – 252.7616	1.420	0.1557
DRB1*14:01	1.25% (1)	3.49%	0.44	0.6812				
DRB1*14:04	2.50% (2)	0.85%	0.35	0.60				
DRB1*14:06	0% (0)	0.04%	0.0072	0.0384	10.2174	0.4130 – 252.7616	1.420	0.1557
**DRB1*14:54**	6.25% (5)	0%	**<0.00001**	**0.00048**	359.6490	19.7070 - 6563.5224	3.972	**0.0001**
DRB1*15:01	10.00% (8)	8.02%	0.52	0.7066				
DRB1*15:02	2.50% (2)	0.80%	0.32	0.5907				
DRB1*15:03	0% (0)	0.08%	0.076	0.2432				
DRB1*15:19	0% (0)	0.25%	0.47	0.6836				
**DRB1*16:01**	16.25% (13)	6.44%	**0.0006**	**0.0144**	2.8177	1.5228 - 5.2137	3.300	**0.0010**
DRB1*16:02	1.25% (1)	0.53%	0.93	0.93				

MG, myasthenia gravis; OR, odds ratio; CI, confidence interval; *p*c, *p* values corrected using Benjamini-Hochberg method; **bold**, significant OR.

Table S2. HLA class I and II (A, B, C and, DRB1) alleles (four-digit level) significantly associated (chi-squared test) after correction with dSNMG and the quantification of these associations

Allele	dSNMG 100% (18)	Control group 100% (2468)	*p*	*p*c	OR	95% CI	Z	*p*

A*02:02	0.00% (0)	0.17%	0.0143	0.037180	12.1057	0.6457 - 226.9418	1.668	0.0954
A*02:06	0.00% (0)	0.12%	0.0011	0.003575	19.0386	0.9495 – 381.7645	1.926	0.0541
**A*02:07**	0.00% (0)	0.04%	**<0.00001**	**0.00004875**	**44.4595**	**1.7531 – 1127.5039**	**2.300**	**0.0214**
**A*02:08**	0.00% (0)	0.04%	**<0.00001**	**0.00004875**	**44.4595**	**1.7531 – 1127.5039**	**2.300**	**0.0214**
A*02:11	0.00% (0)	0.16%	0.0054	0.0150428	14.8018	0.7690 – 284.8976	1.786	0.0741
**A*02:27**	0.00% (0)	0.04%	**<0.00001**	**0.00004875**	**44.4595**	**1.7531 – 1127.5039**	**2.300**	**0.0214**
**A*02:30**	0.00% (0)	0.08%	**0.0001**	**0.000433**	**26.6649**	**2370 – 574.8004**	**2.096**	**0.0361**
**A*03:97**	0.00% (0)	0.04%	**<0.00001**	**0.00004875**	**44.4595**	**1.7531 – 1127.5039**	**2.300**	**0.0214**
**A*24:18**	0.00% (0)	0.04%	**<0.00001**	**0.00004875**	**44.4595**	**1.7531 – 1127.5039**	**2.300**	**0.0214**
A*30:04	0.00% (0)	0.12%	0.0011	0.003575	19.0386	0.9495 – 381.7645	1.926	0.0541
A*34:02	0.00% (0)	0.16%	0.0054	0.0150428	14.8018	0.7690 – 284.8976	1.786	0.0741
**A*68:18N**	0.00% (0)	0.04%	**<0.00001**	**0.00004875**	**44.4595**	**1.7531 – 1127.5039**	**2.300**	**0.0214**
**A*74:01**	0.00% (0)	0.04%	**<0.00001**	**0.00004875**	**44.4595**	**1.7531 – 1127.5039**	**2.300**	**0.0214**
A*74:03	0.00% (0)	0.12%	0.0011	0.003575	19.0386	0.9495 – 381.7645	1.926	0.0541
**A*80:01**	0.00% (0)	0.04%	**<0.00001**	**0.00004875**	**44.4595**	**1.7531 – 1127.5039**	**2.300**	**0.0214**
**B*07:04**	0.00% (0)	0.08%	**0.0001**	**0.000336**	**26.6649**	**2370 – 574.8004**	**2.096**	**0.0361**
B*14:01	0.00% (0)	0.16%	0.0054	0.013292	14.8018	0.7690 – 284.8976	1.786	0.0741
**B*15:03**	0.00% (0)	0.08%	**0.0001**	**0.000336**	**26.6649**	**2370 – 574.8004**	**2.096**	**0.0361**
**B*15:08**	0.00% (0)	0.08%	**0.0001**	**0.000336**	**26.6649**	**2370 – 574.8004**	**2.096**	**0.0361**
**B*15:09**	0.00% (0)	0.04%	**<0.00001**	**0.000064**	**44.4595**	**1.7531 – 1127.5039**	**2.300**	**0.0214**

B*15:18	0.00% (0)	0.16%	0.0054	0.013292	14.8018	0.7690 – 284.8976	1.786	0.0741
**B*15:39**	0.00% (0)	0.04%	**<0.00001**	**0.000064**	**44.4595**	**1.7531 – 1127.5039**	**2.300**	**0.0214**
**B*15:73**	0.00% (0)	0.04%	**<0.00001**	**0.000064**	**44.4595**	**1.7531 – 1127.5039**	**2.300**	**0.0214**
B*18:03	0.00% (0)	0.16%	0.0054	0.013292	14.8018	0.7690 – 284.8976	1.786	0.0741
**B*18:04**	0.00% (0)	0.08%	**0.0001**	**0.000336**	**26.6649**	**2370 – 574.8004**	**2.096**	**0.0361**
**B*27:07**	0.00% (0)	0.08%	**0.0001**	**0.000336**	**26.6649**	**2370 – 574.8004**	**2.096**	**0.0361**
**B*35:14**	0.00% (0)	0.04%	**<0.00001**	**0.000064**	**44.4595**	**1.7531 – 1127.5039**	**2.300**	**0.0214**
**B*39:10**	0.00% (0)	0.04%	**<0.00001**	**0.000064**	**44.4595**	**1.7531 – 1127.5039**	**2.300**	**0.0214**
**B*39:31**	0.00% (0)	0.04%	**<0.00001**	**0.000064**	**44.4595**	**1.7531 – 1127.5039**	**2.300**	**0.0214**
**B*44:04**	0.00% (0)	0.04%	**<0.00001**	**0.000064**	**44.4595**	**1.7531 – 1127.5039**	**2.300**	**0.0214**
**B*44:27**	**5.55% (1)**	**0.00%**	**<0.00001**	**0.000064**	**423.1714**	**16.6558 – 10751.4466**	**3.664**	**0.0002**
**B*46:01**	0.00% (0)	0.08%	**0.0001**	**0.000336**	**26.6649**	**2370 – 574.8004**	**2.096**	**0.0361**
B*48:01	0.00% (0)	0.16%	0.0054	0.013292	14.8018	0.7690 – 284.8976	1.786	0.0741
**B*51:02**	0.00% (0)	0.05%	**<0.00001**	**0.000064**	**44.4595**	**1.7531 – 1127.5039**	**2.300**	**0.0214**
**B*51:05**	0.00% (0)	0.08%	**0.0001**	**0.000336**	**26.6649**	**2370 – 574.8004**	**2.096**	**0.0361**
B*51:07	0.00% (0)	0.13%	0.0011	0.003352	19.0386	0.9495 – 381.7645	1.926	0.0541
..B*51:08	0.00% (0)	0.16%	0.005	0.013292	14.8018	0.7690 – 284.8976	1.786	0.0741
B*51:65	0.00% (0)	0.12%	0.0011	0.003352	19.0386	0.9495 – 381.7645	1.926	0.0541
**B*53:01**	0.00% (0)	0.04%	**<0.00001**	**0.000064**	**44.4595**	**1.7531 – 1127.5039**	**2.300**	**0.0214**
**B*57:02**	0.00% (0)	0.08%	**0.0001**	**0.000336**	**26.6649**	**1.2370 – 574.8004**	**2.096**	**0.0361**

**B*73:01**	0.00% (0)	0.08%	**0.0001**	**0.000336**	**26.6649**	**1.2370 – 574.8004**	**2.096**	**0.0361**
**C*08:01**	0.00% (0)	0.04%	**<0.00001**	**0.00007**	**44.4595**	**1.7531 – 1127.5039**	**2.300**	**0.0214**
C*08:03	0.00% (0)	0.12%	0.0011	0.005133	19.0386	0.9495 – 381.7645	1.926	0.0541
**C*12:12**	0.00% (0)	0.04%	**<0.00001**	**0.00007**	**44.4595**	**1.7531 – 1127.5039**	**2.300**	**0.0214**
C*15:04	0.00% (0)	0.12%	0.0011	0.005133	19.0386	0.9495 – 381.7645	1.926	0.0541
**C*15:06**	0.00% (0)	0.04%	**<0.00001**	**0.00007**	**44.4595**	**1.7531 – 1127.5039**	**2.300**	**0.0214**
**C*18:01**	0.00% (0)	0.04%	**<0.00001**	**0.00007**	**44.4595**	**1.7531 – 1127.5039**	**2.300**	**0.0214**
DRB1*01:03	0.00% (0)	0.12%	0.0011	0.002778	19.0386	0.9495 – 381.7645	1.926	0.0541
**DRB1*01:11**	0.00% (0)	0.04%	**<0.00001**	**0.00006**	**44.4595**	**1.7531 – 1127.5039**	**2.300**	**0.0214**
DRB1*04:08	0.00% (0)	0.16%	0.0054	0.0129600	14.8018	0.7690 – 284.8976	1.786	0.0741
**DRB1*04:10**	0.00% (0)	0.08%	**0.0001**	**0.0003428**	**26.6649**	**2370 – 574.8004**	**2.096**	**0.0361**
**DRB1*08:02**	0.00% (0)	0.08%	**0.0001**	**0.0003428**	**26.6649**	**2370 – 574.8004**	**2.096**	**0.0361**
DRB1*08:03	0.00% (0)	0.12%	0.0011	0.002778	19.0386	0.9495 – 381.7645	1.926	0.0541
DRB1*08:04	0.00% (0)	0.12%	0.0011	0.002778	19.0386	0.9495 – 381.7645	1.926	0.0541
**DRB1*08:17**	0.00% (0)	0.08%	**0.0001**	**0.0003428**	**26.6649**	**2370 – 574.8004**	**2.096**	**0.0361**
DRB1*11:02	0.00% (0)	0.20%	0.0143	0.03268571	12.1057	0.6457 - 226.9418	1.668	0.0954
**DRB1*11:08**	0.00% (0)	0.08%	**0.0001**	**0.0003428**	**26.6649**	**2370 – 574.8004**	**2.096**	**0.0361**
**DRB1*11:11**	0.00% (0)	0.04%	**<0.00001**	**0.00006**	**44.4595**	**1.7531 – 1127.5039**	**2.300**	**0.0214**
DRB1*11:15	0.00% (0)	0.12%	0.0011	0.002778	19.0386	0.9495 – 381.7645	1.926	0.0541
**DRB1*11:33**	0.00% (0)	0.04%	**<0.00001**	**0.00006**	**44.4595**	**1.7531 – 1127.5039**	**2.300**	**0.0214**

**DRB1*11:39**	0.00% (0)	0.04%	**<0.00001**	**0.00006**	**44.4595**	**1.7531 – 1127.5039**	**2.300**	**0.0214**
**DRB1*11:43**	0.00% (0)	0.04%	**<0.00001**	**0.00006**	**44.4595**	**1.7531 – 1127.5039**	**2.300**	**0.0214**
**DRB1*12:02**	0.00% (0)	0.08%	**0.0001**	**0.0003428**	**26.6649**	**1.2370 – 574.8004**	**2.096**	**0.0361**
DRB1*13:01	0.00% (0)	7.78%	0.017	0.0370909	0.0735	0.0045 – 1.1891	1.838	0.0661
DRB1*13:05	0.00% (0)	0.12%	0.0011	0.002778	19.0386	0.9495 – 381.7645	1.926	0.0541
**DRB1*13:22**	0.00% (0)	0.04%	**<0.00001**	**0.00006**	**44.4595**	**1.7531 – 1127.5039**	**2.300**	**0.0214**
**DRB1*14:06**	0.00% (0)	0.04%	**<0.00001**	**0.00006**	**44.4595**	**1.7531 – 1127.5039**	**2.300**	**0.0214**
**DRB1*14:54**	**11.11% (2)**	**0.00%**	**<0.00001**	**0.00006**	**748.0303**	**34.5591 - 16191.0695**	**4.218**	**< 0.0001**
**DRB1*15:03**	0.00% (0)	0.08%	**0.0001**	**0.0003428**	**26.6649**	**2370 - 574,8004**	**2.096**	**0.0361**

MG, myasthenia gravis; dSNMG, double-seronegative MG; OR, odds ratio; CI, confidence interval; *p*c, *p* values corrected using Benjamini-Hochberg method; **bold**, significant OR.

Table S3. Linkage disequilibrium in the MG group and the control group.

MG cases – HLA-A haplotype

HLA-A haplotype	Observed frequency (%)	Theorethical frequency (%)	LD
A*02-A*24	15.00	5.25	9.75
A*01-A*03	7.50	1.22	6.28
A*01-A*02	10.00	5.69	4.31
A*01-A*32	5.00	1.02	3.98
A*02-A*26	5.00	1.75	3.25
A*01-A*24	5.00	2.44	2.56
A*23-A*68	2.50	0.02	2.48
A*31-A*32	2.50	0.08	2.42
A*25-A*33	2.50	0.09	2.41
A*03-A*33	2.50	0.19	2.31

**A*26-A*26**	**2.50**	**0.25**	**2.25**
**A*11-A*24**	**2.50**	**0.38**	**2.13**
**A*02-A*29**	**2.50**	**0.44**	**2.06**
**A*02-A*30**	**2.50**	**0.44**	**2.06**
**A*03-A*32**	**2.50**	**0.47**	**2.03**
A*24-A*25	2.50	0.56	1.94
A*02-A*11	2.50	0.88	1.63
A*02-A*25	2.50	1.31	1.19
A*02-A*32	2.50	2.19	0.31
A*02-A*02	12.50	12.25	0.25
A*24-A*24	2.50	2.25	0.25
A*02-A*03	2.50	2.63	-0.13
A*01-A*01	2.50	2.64	-0.14

LD, linkage disequilibrium.

MG cases – HLA-B haplotype

**HLA-B haplotype**	**Observed frequency (%)**	**Theorethical frequency (%)**	**LD**
**B*40-B*47**	**5.00**	**0.25**	**4.75**
**B*07-B*35**	**5.00**	**0.52**	**4.48**
**B*18-B*40**	**5.00**	**1.13**	**3.88**
**B*35-B*44**	**5.00**	**1.20**	**3.80**
**B*08-B*44**	**5.00**	**1.31**	**3.69**
**B*35-B*40**	**5.00**	**1.38**	**3.63**
**B*08-B*18**	**5.00**	**1.69**	**3.31**
**B*38-B*57**	**2.50**	**0.09**	**2.41**
**B*27-B*57**	**2.50**	**0.13**	**2.38**
**B*51-B*58**	**2.50**	**0.13**	**2.38**
**B*35-B*52**	**2.50**	**0.17**	**2.33**
**B*08-B*55**	**2.50**	**0.19**	**2.31**
**B*08-B*73**	**2.50**	**0.19**	**2.31**
**B*13-B*27**	**2.50**	**0.19**	**2.31**
**B*15-B*18**	**2.50**	**0.28**	**2.22**

**B*18-B*39**	**2.50**	**0.28**	**2.22**
**B*07-B*44**	**2.50**	**0.33**	**2.17**
**B*08-B*15**	**2.50**	**0.38**	**2.13**
**B*08-B*39**	**2.50**	**0.38**	**2.13**
**B*13-B*51**	**2.50**	**0.38**	**2.13**
**B*18-B*38**	**2.50**	**0.42**	**2.08**
**B*27-B*51**	**2.50**	**0.50**	**2.00**
B*13-B*35	2.50	0.52	1.98
B*08-B*38	2.50	0.56	1.94
B*27-B*35	2.50	0.69	1.81
B*44-B*51	2.50	0.88	1.63
B*18-B*44	2.50	0.98	1.52
B*40-B*51	2.50	1.00	1.50
B*18-B*51	2.50	1.13	1.38
B*35-B*51	2.50	1.38	1.13
B*08-B*40	2.50	1.50	1.00
B*08-B*51	2.50	1.50	1.00
B*08-B*35	2.50	2.06	0.44

LD, linkage disequilibrium.

MG cases – HLA-DRB1 haplotype

HLA-DRB1 haplotype	Observed frequency (%)	Theorethical frequency (%)	LD
**DRB1*03-DRB1*16**	**10.00**	**1.75**	**8.25**
**DRB1*11-DRB1*16**	**10.00**	**3.06**	**6.94**
**DRB1*04-DRB1*15**	**7.50**	**1.09**	**6.41**
**DRB1*04-DRB1*16**	**7.50**	**1.53**	**5.97**
**DRB1*01-DRB1*07**	**5.00**	**0.84**	**4.16**
**DRB1*03-DRB1*14**	**5.00**	**1.00**	**4.00**
**DRB1*01-DRB1*14**	**5.00**	**1.13**	**3.88**
**DRB1*07-DRB1*11**	**5.00**	**1.31**	**3.69**
**DRB1*01-DRB1*11**	**5.00**	**1.97**	**3.03**

**DRB1*11-DRB1*15**	**5.00**	**2.19**	**2.81**
**DRB1*04-DRB1*13**	**2.50**	**0.11**	**2.39**
**DRB1*11-DRB1*12**	**2.50**	**0.22**	**2.28**
**DRB1*10-DRB1*14**	**2.50**	**0.25**	**2.25**
DRB1*07-DRB1*14	2.50	0.75	1.75
DRB1*07-DRB1*15	2.50	0.94	1.56
DRB1*01-DRB1*03	2.50	1.13	1.38
DRB1*03-DRB1*15	2.50	1.25	1.25
DRB1*14-DRB1*15	2.50	1.25	1.25
DRB1*01-DRB1*15	2.50	1.41	1.09
DRB1*03-DRB1*11	2.50	1.75	0.75
DRB1*14-DRB1*16	2.50	1.75	0.75
DRB1*01-DRB1*16	2.50	1.97	0.53
DRB1*15-DRB1*16	2.50	2.19	0.31
DRB1*11-DRB1*11	2.50	3.06	-0.56

LD, linkage disequilibrium.

Controls - HLA-A haplotype

**HLA-A haplotype**	**Observed frequency (%)**	**Theorethical frequency (%)**	**LD**
**A*01-A*02**	**10.46**	**4.09**	**6.37**
**A*02-A*03**	**6.43**	**2.59**	**3.84**
**A*02-A*24**	**6.17**	**3.36**	**2.81**
**A*01-A*24**	**4.29**	**1.85**	**2.44**
A*02-A*11	4.02	2.04	1.98
A*24-A*32	1.88	0.53	1.35
A*02-A*26	2.95	1.60	1.34
A*03-A*26	1.88	0.56	1.32
A*03-A*24	2.41	1.17	1.24
A*02-A*68	2.14	0.95	1.20
A*24-A*25	1.61	0.43	1.18
A*03-A*11	1.88	0.71	1.16

A*02-A*32	2.14	1.17	0.98
A*02-A*33	1.34	0.40	0.94
A*26-A*31	1.07	0.17	0.91
A*24-A*26	1.61	0.73	0.88
A*03-A*68	1.07	0.33	0.74
A*01-A*23	1.07	0.36	0.71
A*31-A*32	0.80	0.12	0.68
A*23-A*26	0.80	0.14	0.66
A*26-A*68	0.80	0.21	0.60
A*01-A*25	1.07	0.52	0.55
A*11-A*25	0.80	0.26	0.54
A*11-A*11	1.07	0.56	0.51
A*29-A*68	0.54	0.04	0.49
A*02-A*30	1.07	0.58	0.49
A*01-A*11	1.61	1.13	0.48
A*11-A*66	0.54	0.06	0.48
A*23-A*25	0.54	0.08	0.45
A*11-A*29	0.54	0.09	0.45
A*02-A*23	1.07	0.66	0.42
A*26-A*30	0.54	0.13	0.41
A*02-A*25	1.34	0.95	0.39
A*24-A*68	0.80	0.43	0.37
A*24-A*24	1.88	1.52	0.36
A*01-A*29	0.54	0.18	0.36
A*11-A*23	0.54	0.18	0.36
A*02-A*31	1.07	0.77	0.31
A*26-A*32	0.54	0.25	0.28
A*01-A*68	0.80	0.52	0.28
A*24-A*30	0.54	0.26	0.27
A*25-A*34	0.27	0.00	0.26
A*01-A*36	0.27	0.02	0.25
A*01-A*69	0.27	0.02	0.25
A*29-A*30	0.27	0.03	0.24
A*25-A*66	0.27	0.03	0.24

A*30-A*33	0.27	0.03	0.24
A*29-A*31	0.27	0.03	0.23
A*32-A*66	0.27	0.03	0.23
A*33-A*68	0.27	0.05	0.22
A*01-A*30	0.54	0.32	0.21
A*11-A*32	0.54	0.32	0.21
A*30-A*31	0.27	0.06	0.21
A*32-A*33	0.27	0.06	0.20
A*03-A*25	0.54	0.33	0.20
A*23-A*31	0.27	0.07	0.20
A*30-A*68	0.27	0.07	0.19
A*31-A*31	0.27	0.08	0.19
A*01-A*26	1.07	0.89	0.19
A*01-A*03	1.61	1.43	0.18
A*24-A*66	0.27	0.10	0.17
A*23-A*32	0.27	0.10	0.16
A*01-A*32	0.80	0.64	0.16
A*01-A*01	2.41	2.25	0.16
A*11-A*33	0.27	0.11	0.16
A*03-A*29	0.27	0.11	0.15
A*25-A*25	0.27	0.12	0.15
A*03-A*32	0.54	0.41	0.13
A*01-A*31	0.54	0.42	0.11
A*11-A*30	0.27	0.16	0.11
A*11-A*26	0.54	0.44	0.09
A*24-A*33	0.27	0.18	0.09
A*32-A*32	0.27	0.18	0.08
A*03-A*30	0.27	0.20	0.06
A*11-A*31	0.27	0.21	0.06
A*02-A*66	0.27	0.22	0.05
A*01-A*33	0.27	0.22	0.05
A*03-A*23	0.27	0.23	0.04
A*11-A*68	0.27	0.26	0.01
A*03-A*31	0.27	0.27	0.00

A*24-A*31	0.27	0.35	-0.08
A*03-A*03	0.80	0.91	-0.10
A*11-A*24	0.80	0.93	-0.12
A*02-A*02	6.97	7.40	-0.43

LD, linkage disequilibrium.

Controls - HLA-B haplotype

HLA-B haplotype	Observed frequency (%)	Theorethical frequency (%)	LD
**B*15-B*35**	**2.96**	**0.76**	**2.21**
B*08-B*18	2.70	0.82	1.87
B*18-B*51	2.70	0.92	1.77
B*08-B*14	1.62	0.17	1.45
B*15-B*18	1.89	0.52	1.36
B*18-B*44	2.16	0.98	1.18
B*15-B*44	1.62	0.46	1.15
B*07-B*27	1.35	0.27	1.08
B*07-B*18	1.62	0.58	1.04
B*13-B*51	1.35	0.37	0.98
B*08-B*27	1.35	0.38	0.97
B*35-B*40	1.62	0.66	0.96
B*40-B*44	1.35	0.40	0.95
B*35-B*52	1.35	0.41	0.94
B*35-B*35	3.23	2.32	0.92
B*38-B*51	1.35	0.45	0.90
B*18-B*40	1.35	0.45	0.89
B*35-B*41	1.08	0.21	0.87
B*38-B*44	1.35	0.48	0.87
B*07-B*44	1.35	0.51	0.83
B*18-B*35	2.43	1.60	0.82
B*35-B*51	2.16	1.33	0.82
B*07-B*35	1.62	0.84	0.78

B*39-B*58	0.81	0.05	0.75
B*08-B*40	1.08	0.34	0.74
B*35-B*44	2.16	1.42	0.74
B*38-B*55	0.81	0.07	0.74
B*15-B*49	0.81	0.11	0.70
B*08-B*35	1.89	1.19	0.70
B*27-B*39	0.81	0.13	0.68
B*08-B*51	1.35	0.68	0.66
B*07-B*52	0.81	0.15	0.66
B*49-B*51	0.81	0.19	0.62
B*08-B*39	0.81	0.21	0.60
B*13-B*38	0.81	0.21	0.59
B*07-B*51	1.08	0.48	0.59
B*07-B*13	0.81	0.23	0.58
B*27-B*38	0.81	0.25	0.56
B*44-B*52	0.81	0.25	0.56
B*18-B*38	1.08	0.54	0.54
B*44-B*51	1.35	0.81	0.53
B*51-B*73	0.54	0.02	0.52
B*27-B*47	0.54	0.04	0.50
B*08-B*13	0.81	0.33	0.48
B*35-B*49	0.81	0.33	0.48
B*13-B*14	0.54	0.09	0.45
B*40-B*51	0.81	0.38	0.43
B*35-B*56	0.54	0.12	0.42
B*35-B*57	0.54	0.16	0.37
B*13-B*18	0.81	0.44	0.37
B*13-B*40	0.54	0.18	0.36
B*08-B*44	1.08	0.73	0.35
B*44-B*58	0.54	0.19	0.35
B*14-B*44	0.54	0.20	0.34
B*44-B*49	0.54	0.20	0.34
B*35-B*55	0.54	0.21	0.33
B*13-B*15	0.54	0.21	0.33

B*14-B*18	0.54	0.23	0.31
B*39-B*51	0.54	0.24	0.30
B*35-B*38	1.08	0.78	0.30
B*39-B*44	0.54	0.25	0.29
B*27-B*45	0.27	0.01	0.26
B*07-B*38	0.54	0.28	0.26
B*18-B*52	0.54	0.28	0.26
B*50-B*52	0.27	0.01	0.26
B*37-B*55	0.27	0.02	0.25
B*37-B*49	0.27	0.03	0.24
B*15-B*50	0.27	0.03	0.24
B*49-B*55	0.27	0.03	0.24
B*13-B*47	0.27	0.03	0.24
B*40-B*56	0.27	0.03	0.23
B*35-B*58	0.54	0.31	0.23
B*27-B*56	0.27	0.04	0.23
B*15-B*47	0.27	0.04	0.23
B*58-B*58	0.27	0.04	0.23
B*38-B*56	0.27	0.04	0.23
B*08-B*50	0.27	0.04	0.23
B*50-B*51	0.27	0.05	0.22
B*27-B*57	0.27	0.05	0.22
B*37-B*40	0.27	0.05	0.22
B*15-B*57	0.27	0.05	0.22
B*38-B*57	0.27	0.06	0.21
B*44-B*44	1.08	0.86	0.21
B*13-B*41	0.27	0.06	0.21
B*49-B*52	0.27	0.06	0.21
B*27-B*37	0.27	0.06	0.21
B*37-B*38	0.27	0.06	0.21
B*08-B*56	0.27	0.06	0.21
B*27-B*41	0.27	0.07	0.20
B*07-B*37	0.27	0.07	0.20
B*38-B*41	0.27	0.07	0.20

B*39-B*39	0.27	0.07	0.20
B*07-B*41	0.27	0.07	0.20
B*07-B*55	0.27	0.07	0.20
B*08-B*57	0.27	0.08	0.19
B*13-B*58	0.27	0.08	0.19
B*18-B*47	0.27	0.09	0.18
B*40-B*58	0.27	0.09	0.18
B*13-B*49	0.27	0.09	0.18
B*51-B*57	0.27	0.09	0.18
B*13-B*35	0.81	0.64	0.17
B*27-B*58	0.27	0.10	0.17
B*44-B*57	0.27	0.10	0.17
B*14-B*27	0.27	0.10	0.16
B*08-B*41	0.27	0.11	0.16
B*37-B*51	0.27	0.11	0.16
B*14-B*38	0.27	0.11	0.16
B*07-B*58	0.27	0.11	0.16
B*39-B*40	0.27	0.12	0.15
B*40-B*52	0.27	0.12	0.15
B*51-B*55	0.27	0.12	0.15
B*07-B*14	0.27	0.12	0.15
B*35-B*47	0.27	0.12	0.15
B*44-B*55	0.27	0.13	0.14
B*18-B*37	0.27	0.13	0.14
B*27-B*52	0.27	0.13	0.14
B*15-B*39	0.27	0.13	0.14
B*15-B*52	0.27	0.13	0.14
B*18-B*41	0.27	0.14	0.13
B*07-B*39	0.27	0.15	0.12
B*27-B*51	0.54	0.43	0.11
B*07-B*08	0.54	0.43	0.11
B*51-B*58	0.27	0.18	0.09
B*35-B*37	0.27	0.18	0.08
B*27-B*35	0.81	0.74	0.07

B*27-B*40	0.27	0.21	0.06
B*08-B*52	0.27	0.21	0.06
B*18-B*58	0.27	0.21	0.06
B*38-B*40	0.27	0.22	0.05
B*18-B*49	0.27	0.23	0.04
B*51-B*52	0.27	0.24	0.03
B*18-B*27	0.54	0.51	0.03
B*15-B*27	0.27	0.24	0.03
B*38-B*38	0.27	0.26	0.01
B*18-B*39	0.27	0.28	-0.01
B*14-B*35	0.27	0.33	-0.06
B*13-B*44	0.27	0.39	-0.12
B*08-B*15	0.27	0.39	-0.12
B*08-B*38	0.27	0.40	-0.13
B*35-B*39	0.27	0.41	-0.14
B*15-B*51	0.27	0.44	-0.17
B*27-B*44	0.27	0.45	-0.18
B*51-B*51	0.54	0.77	-0.23
B*08-B*08	0.27	0.61	-0.34
B*18-B*18	0.54	1.11	-0.57

LD, linkage disequilibrium.

Controls - HLA-DRB1 haplotype

**HLA-DRB1 haplotype**	**Observed frequency (%)**	**Theorethical frequency (%)**	**LD**
**DRB1*11-DRB1*13**	**6.72**	**2.36**	**4.36**
**DRB1*04-DRB1*11**	**5.97**	**2.21**	**3.76**
**DRB1*11-DRB1*16**	**5.22**	**1.84**	**3.38**
**DRB1*04-DRB1*07**	**4.48**	**1.29**	**3.18**
**DRB1*01-DRB1*03**	**3.73**	**0.75**	**2.98**
**DRB1*07-DRB1*11**	**5.22**	**2.29**	**2.94**
**DRB1*11-DRB1*15**	**4.48**	**1.77**	**2.71**

**DRB1*14-DRB1*16**	**2.99**	**0.42**	**2.57**
**DRB1*07-DRB1*13**	**3.73**	**1.38**	**2.35**
DRB1*03-DRB1*15	2.99	1.00	1.98
DRB1*03-DRB1*14	2.24	0.50	1.74
DRB1*04-DRB1*13	2.99	1.34	1.65
DRB1*01-DRB1*13	2.24	0.80	1.44
DRB1*15-DRB1*16	2.24	0.84	1.40
DRB1*12-DRB1*15	1.49	0.20	1.29
DRB1*12-DRB1*16	1.49	0.21	1.28
DRB1*03-DRB1*16	2.24	1.04	1.19
DRB1*03-DRB1*03	2.24	1.25	0.99
DRB1*13-DRB1*14	1.49	0.53	0.96
DRB1*03-DRB1*07	2.24	1.29	0.94
DRB1*01-DRB1*11	2.24	1.33	0.91
DRB1*13-DRB1*13	2.24	1.43	0.81
DRB1*03-DRB1*11	2.99	2.21	0.77
DRB1*01-DRB1*04	1.49	0.75	0.74
DRB1*04-DRB1*09	0.75	0.04	0.70
DRB1*08-DRB1*14	0.75	0.05	0.70
DRB1*15-DRB1*15	1.49	0.80	0.69
DRB1*01-DRB1*08	0.75	0.08	0.67
DRB1*10-DRB1*15	0.75	0.10	0.65
DRB1*10-DRB1*16	0.75	0.10	0.64
DRB1*07-DRB1*08	0.75	0.13	0.62
DRB1*07-DRB1*10	0.75	0.13	0.62
DRB1*04-DRB1*15	1.49	1.00	0.49
DRB1*12-DRB1*13	0.75	0.27	0.48
DRB1*04-DRB1*16	1.49	1.04	0.45
DRB1*01-DRB1*14	0.75	0.30	0.45
DRB1*11-DRB1*12	0.75	0.44	0.30
DRB1*04-DRB1*04	1.49	1.25	0.24
DRB1*07-DRB1*14	0,75	0.52	0.23
DRB1*07-DRB1*07	1.49	1.34	0.15
DRB1*01-DRB1*15	0.75	0.60	0.14

DRB1*01-DRB1*16	0.75	0.63	0.12
DRB1*01-DRB1*07	0.75	0.78	-0.03
DRB1*07-DRB1*15	0.75	1.04	-0.29
DRB1*07-DRB1*16	0.75	1.08	-0.33
DRB1*13-DRB1*16	0.75	1.11	-0.37
DRB1*03-DRB1*04	0.75	1.25	-0.51
DRB1*03-DRB1*13	0.75	1.34	-0.59
DRB1*11-DRB1*11	2.99	3.91	-0.93

LD, linkage disequilibrium.
